# Skin-to-skin contact for the prevention of neonatal hypoglycaemia: a systematic review and meta-analysis

**DOI:** 10.1186/s12884-023-06057-8

**Published:** 2023-10-21

**Authors:** Libby G. Lord, Jane E. Harding, Caroline A. Crowther, Luling Lin

**Affiliations:** Liggins Institute, University of Auckland, 85 Park Road, Grafton, Auckland, 1023 New Zealand

**Keywords:** Hypoglycaemia, Skin-to-skin, Infant, Newborn, Kangaroo Mother Care, Kangaroo care, Neonatology

## Abstract

**Background:**

Skin-to-skin contact between mother and infant after birth is recommended to promote breastfeeding and maternal-infant bonding. However, its impact on the incidence of neonatal hypoglycaemia is unknown. We conducted a systematic review and meta-analysis to assess this.

**Methods:**

Published randomised control trials (RCTs), quasi-RCTs, non-randomised studies of interventions, cohort, or case–control studies with an intervention of skin-to-skin care compared to other treatment were included without language or date restrictions. The primary outcome was neonatal hypoglycaemia (study-defined). We searched 4 databases and 4 trial registries from inception to May 12^th^, 2023. Quality of studies was assessed using Cochrane Risk of Bias 1 or Effective Public Health Practice Project Quality Assessment tools. Certainty of evidence was assessed using the Grading of Recommendations Assessment, Development and Evaluation (GRADE) approach. Results were synthesised using RevMan 5.4.1 or STATA and analysed using random-effects meta-analyses where possible, otherwise with direction of findings tables. This review was registered prospectively on PROSPERO (CRD42022328322).

**Results:**

This review included 84,900 participants in 108 studies, comprising 65 RCTs, 16 quasi-RCTs, seven non-randomised studies of intervention, eight prospective cohort studies, nine retrospective cohort studies and three case–control studies. Evidence suggests skin-to-skin contact may result in a large reduction in the incidence of neonatal hypoglycaemia (7 RCTs/quasi-RCTs, 922 infants, RR 0.29 (0.13, 0.66), *p* < 0.0001, I^2^ = 47%). Skin-to-skin contact may reduce the incidence of admission to special care or neonatal intensive care nurseries for hypoglycaemia (1 observational study, 816 infants, OR 0.50 (0.25–1.00), *p* = 0.050), but the evidence is very uncertain. Skin-to-skin contact may reduce duration of initial hospital stay after birth (31 RCTs, 3437 infants, MD -2.37 (-3.66, -1.08) days, *p* = 0.0003, I^2^ = 90%, *p* for Egger’s test = 0.02), and increase exclusive breastmilk feeding from birth to discharge (1 observational study, 1250 infants, RR 4.30 (3.19, 5.81), *p* < 0.0001), but the evidence is very uncertain.

**Conclusion:**

Skin-to-skin contact may lead to a large reduction in the incidence of neonatal hypoglycaemia. This, along with other established benefits, supports the practice of skin-to-skin contact for all infants and especially those at risk of hypoglycaemia.

**Supplementary Information:**

The online version contains supplementary material available at 10.1186/s12884-023-06057-8.

## Introduction

Neonatal hypoglycaemia affects up to 5–15% of infants [1], and is associated with poor neurodevelopmental outcomes [2]. Therefore, prevention of neonatal hypoglycaemia is crucial to improve health outcomes for at-risk infants, including those born preterm, small for gestational age, large for gestational age or to mothers with diabetes [3].

Skin-to-skin contact involves the naked infant being placed prone on the bare chest of the mother soon after birth [4]. The UNICEF Baby Friendly Initiative Guidelines suggest the duration of skin-to-skin contact should be a minimum of one hour or until the first feed is complete [5]. Sometimes, the infant is placed in skin-to-skin contact with the father or another caregiver. The practice is also a component of Kangaroo Care and is now recognised to have many benefits for both caregivers and infants, including promoting physiological stability in infants [4], promoting early and exclusive breastfeeding and parent-infant bonding [4, 6]. Kangaroo Mother Care (KMC) specifically refers to extended skin-to-skin contact (at least 8 h per day) for preterm and low birthweight infants (< 2.5 kg), in combination with exclusive breastfeeding support[7]. In these infants, the World Health Organization (WHO) recommend immediate initiation of KMC after birth. Uptake of KMC in low-resource settings has helped improve health outcomes, especially when incubators are unavailable [8].

There are several mechanisms through which skin-to-skin contact may potentially reduce the incidence of neonatal hypoglycaemia, including reducing the infant’s energy expenditure by promoting thermoregulation [6], increasing quiet sleep time [9] and reducing crying [10]. Skin-to-skin contact also promotes early breastfeeding initiation [11] which provides crucial nutrition to the infant.

Although skin-to-skin contact is recommended for inclusion in neonatal hypoglycaemia prevention guidelines by the UNICEF Baby Friendly Initiative [12], there is limited data about the efficacy of skin-to-skin contact for preventing neonatal hypoglycaemia. We undertook a systematic review to examine whether skin-to-skin contact is effective in preventing neonatal hypoglycaemia compared to standard care or other treatments, with the aim of informing future guideline development and clinical decision-making.

## Methods

This review was reported according to Preferred Reporting Items for Systematic Reviews and Meta-Analyses (PRISMA) guidelines [13] (Additional file 1) and registered prospectively in PROSPERO (registration number CRD42022328322). The systematic review protocol is included as an additional file (Additional file 2).

### Search strategy and selection criteria

We searched Ovid MEDLINE, Embase, CINAHL Complete and the Cochrane Central Register of Controlled Trials (CENTRAL) from inception to May 12^th^, 2023. We also searched for registered trials in Current Controlled Trials (www.controlled-trials.com.), Clinical Trials [14], Australian and New Zealand Clinical Trials Registry [15] and WHO International Clinical Trials Registry Platform (ICTRP) Search Portal [16] (Additional file 3). Conference abstracts were included if they provided usable summary data.

Inclusion criteria were published randomised controlled trials (RCTs), quasi-RCTs, non-randomised studies of interventions, cohort or case–control studies without restrictions on publication date or language involving women and their infants where the intervention was standard care with skin-to-skin contact (study defined) commenced any time during initial hospitalisation after birth and the comparator was standard care or other treatment without skin-to-skin contact (control). We excluded trials that compared newly introduced skin-to-skin contact to historical standard care data as we were not able to ascertain what other differences in practice occurred between the two time periods. We only included studies with skin-to-skin contact occurring in the comparator group if this was after relevant outcomes were recorded.

The primary outcome was neonatal hypoglycaemia (study-defined). Secondary outcomes were hypoglycaemia (any blood glucose concentration < 2.6 mmol/L) during the initial hospital stay after birth, receipt of treatment for hypoglycaemia (study‐defined, including oral dextrose gel, intravenous dextrose, or other drug therapy) during initial hospital stay, number of episodes of hypoglycaemia (study‐defined), severity of hypoglycaemia (any blood glucose concentration < 2.0 mmol/L or study-defined), admission to special care nursery or neonatal intensive care nursery, admission to special care nursery or neonatal intensive care nursery for hypoglycaemia, hypoglycaemic injury on brain imaging, hyperthermia (study‐defined), hypothermia (study‐defined), duration of initial hospital stay after birth, breastmilk feeding exclusively from birth to discharge, breastmilk feeding exclusively at discharge, adverse effects (study-defined). For studies that reported breastfeeding outcomes at multiple time points that fit within a single analysis window, we used the time point with the highest follow-up rate, or if the follow-up rate was the same, we used the latest time point. For studies that reported multiple temperature or blood glucose measurements, or prevalence of neonatal hypoglycaemia at specific times, we used the data closest to the end of the intervention period.

### Data collection and analysis

Two reviewers (LL and LGL) independently screened titles and abstracts of identified records, assessed potentially eligible full-text articles for inclusion and extracted data into a pre-specified data extraction form using Covidence [17]. In addition to the primary and secondary outcomes, data were also collected on study setting, inclusion and exclusion criteria, funding sources, authors’ declaration of interest, ethics approval, trial registration, details of the intervention and comparator and baseline characteristics of intervention and comparison groups. Because equity for indigenous populations (in our context New Zealand Māori) is a critical part of any health research, we also assessed whether there were any data specifically from indigenous populations, especially Māori. Two independent reviewers (LL and LGL) assessed the risk of bias for included studies using the Cochrane risk of bias 1 tool [18] (RoB 1) for RCTs and quasi-RCTs, and the Effective Public Health Practice Project (EPHPP) Quality Assessment Tool for Quantitative Studies for non-randomised studies, cohort and case–control studies [19]. RoB 1 assesses sequence generation, allocation concealment, blinding of participants and personnel, blinding of outcome assessors, incomplete outcome data, selective reporting and other sources of bias and does not make an overall risk of bias judgement [18]. The EPHPP Quality Assessment Tool assesses selection bias, study design, confounding, blinding, data collection methods and withdrawals and drop-outs, combining these into an overall assessment of risk of bias [20]. Discrepancies in any step were resolved by discussion or with a third author (JH). Abstracts or articles requiring translation were translated by a colleague where possible and otherwise by Google Translate [21].

We assessed certainty of evidence for each key outcome using the Grading of Recommendations Assessment, Development and Evaluation (GRADE) approach [22] and created a “Summary of Findings” table using the Grade Pro Guideline Development Tool (GDT) [23]. The outcomes included for GRADE assessment were neonatal hypoglycaemia (study-defined), receipt of treatment for hypoglycaemia during initial hospital stay after birth, special care nursery or neonatal intensive care nursery admission for hypoglycaemia, hypoglycaemic injury on brain imaging, duration of initial hospital stay after birth, and breastmilk feeding exclusively from birth to discharge.

### Statistical analysis

We undertook meta-analyses using RevMan 5.4.1 [24] using random-effects models and calculated relative risks (RRs) or odds ratios (ORs) for dichotomous outcomes with 95% confidence intervals (CIs). We calculated mean differences (MDs) for continuous outcomes with 95% CIs. We included studies in the meta-analysis that reported raw data for an outcome in the same way as at least one other study. STATA 17.0 [25] was used for pooling the adjusted odds ratios from cohort and case–control studies. *p* < 0.05 denoted statistical significance for all models. We estimated the values for the mean and standard deviation for the studies that provided minimum, maximum and median or lower quartile, median and upper quartile so that the data could be combined in meta-analysis [26]. WebPlotDigitiser [27] was used to read numerical values off graphs for use in meta-analysis. We calculated *I*^2^ and *χ*^2^ to determine statistical heterogeneity, with *I*^2^ > 50% and P < 0.10 in the *χ*^2^ test considered significant heterogeneity. Where significant heterogeneity was observed, we explored the possible causes in subgroup analyses of RCT and quasi-RCT evidence. We planned to conduct sensitivity analyses for outcomes with significant heterogeneity by including only high-quality studies. We assessed publication bias by visual inspection of funnel plots when there were more than 10 trials. Where asymmetry was present, we considered and discussed possible reasons for it. We planned to conduct subgroup analyses to see if the effect of skin-to-skin contact differed for duration of skin-to-skin contact (< 60 min versus ≥ 60 min), timing of initiation (immediate < 10 min after birth versus early 10 min—24 h [28] versus > 24 h to discharge [29]), infants born preterm versus at term, infants at risk of hypoglycaemia versus not at risk, single versus multiple birth, vaginal birth versus caesarean birth and skin-to-skin contact with mother versus skin-to-skin contact with another person. To decide which studies were eligible for each synthesis, we included details of each study in the characteristics of studies table and compared these to our pre-specified groups. All analyses were pre-planned unless otherwise specified.

## Results

**Fig. 1:**
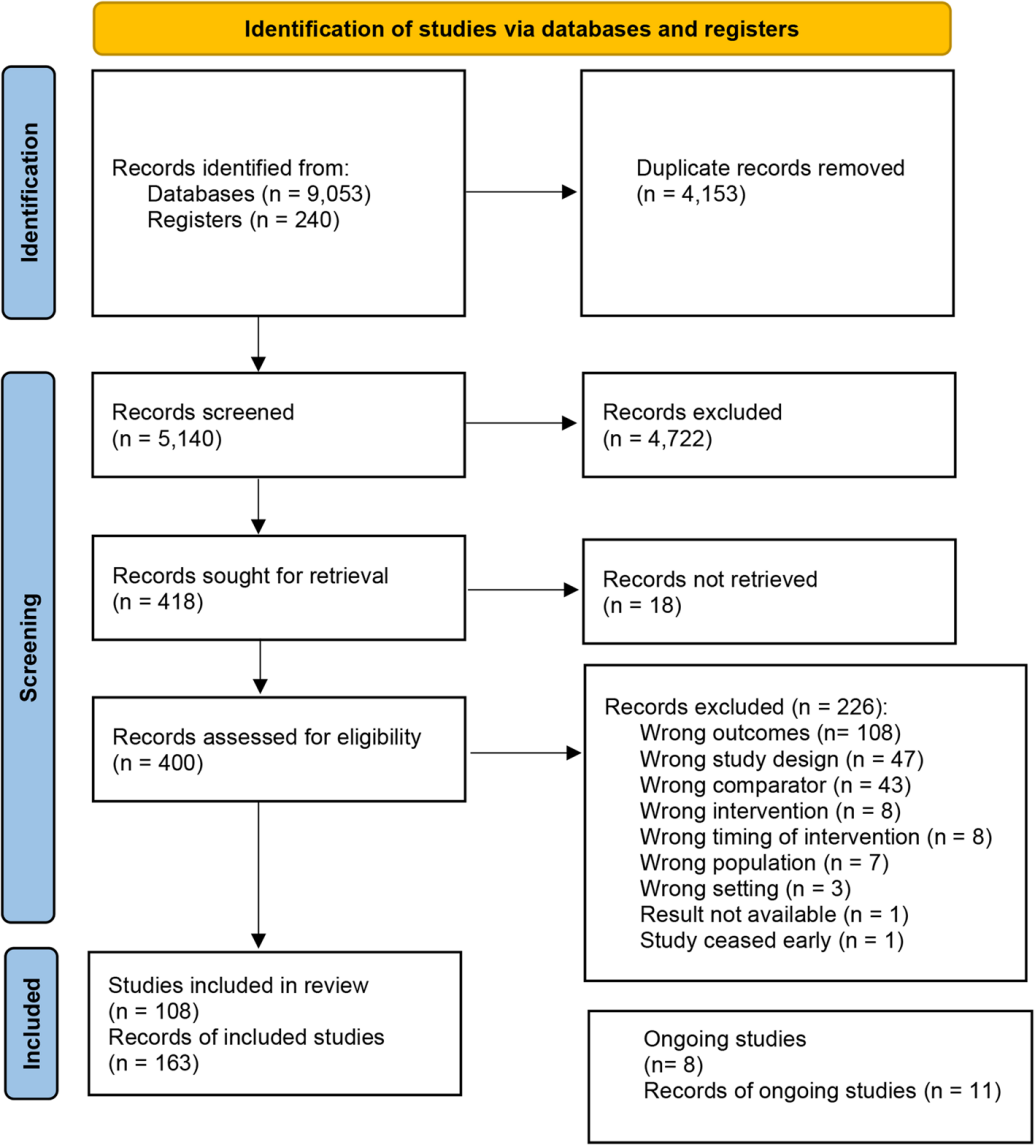
Flow diagram of the included studies

In total, 9293 records were identified from searching. After removing duplicates, we conducted title and abstract screening for 5140 records, followed by full-text screening for 400 records. Eighteen records could not be retrieved. Ultimately, our inclusion criteria were met by a total of 116 studies (163 records). Among those, eight are ongoing studies and 108 studies were included in the review (Fig. 1).

Among the included studies, there were 65 RCTs, 16 quasi-RCTs, seven non-randomised studies of intervention, eight prospective cohort studies, nine retrospective cohort studies and three case–control studies (Additional file 4). The studies were conducted between 1978 and 2021. For RCTs or quasi-RCTs, according to the 2022 World Bank Classification [30], 25 were conducted in high-income countries, 26 in upper-middle-income countries, 26 in lower-middle-income countries and 4 in low-income countries. Among the other study designs, 11 were conducted in high-income countries, 11 in upper-middle-income countries, 1 in a lower-middle-income country and 4 in low-income countries. There were no data specifically reporting indigenous populations.

### Risk of bias or quality of included studies

**Fig. 2:**
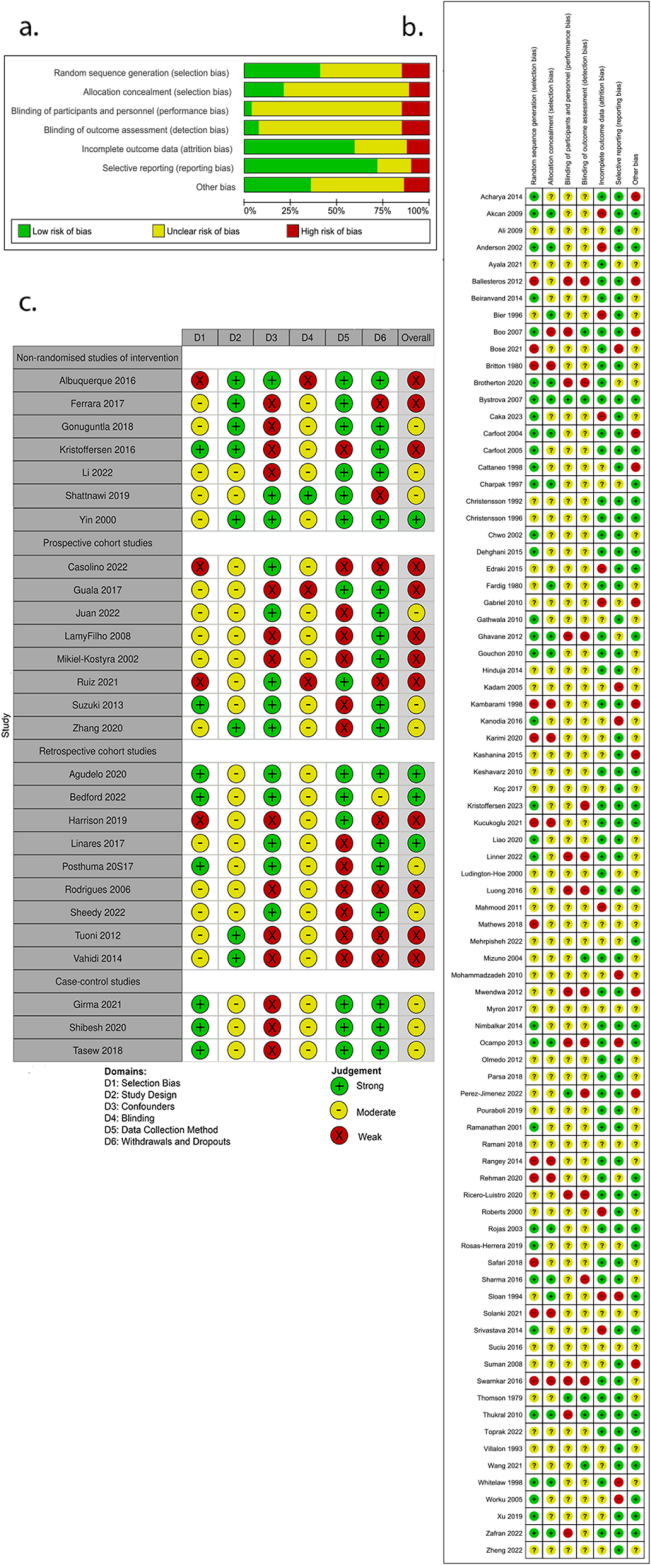
Risk of bias assessment or quality assessment. **a** Risk of bias graph using Cochrane risk of bias tool I: review authors’ judgements about each risk of bias item presented as percentages across all included studies. **b** Risk of bias summary using Cochrane risk of bias tool I: review authors’ judgements about each risk of bias item for each included study. **c** Quality assessment using Effective Public Health Practice Project

The overall methodological quality of the included studies was low (Fig. 2). Among the RCTs or quasi-RCTs, 47/81 (58%) studies had an unclear risk of selection bias due to insufficient information regarding sequence generation, and 12/81 (15%) were at high risk of selection bias because of quasi-randomisation, 63/81 (78%) had an unclear risk of detection bias and 11/81 (14%) had a high risk of detection bias. Although blinding participants was challenging given the nature of the intervention, many studies did not report whether the outcome assessment was blinded. Out of 81 studies, 10 (12%) had a high risk of attrition bias due to loss to follow-up and 8 (10%) were at high risk of reporting bias because some pre-specified outcomes were not reported. In addition, 11/82 (14%) studies had a high risk of other bias due to baseline group imbalance, and 41/81 (51%) had an unclear risk of other bias, mainly due to the unclear role of the funding source. Of the 27 studies with other study designs, four were rated as having strong overall quality, 11 were rated as having moderate quality, mostly due to weak methodology in confounder adjustment or data collection methods domains, and 12 were rated as having weak overall quality due to weak methodology in more than one domain.

### Primary outcome: Neonatal hypoglycaemia (study-defined)

**Fig. 3:**
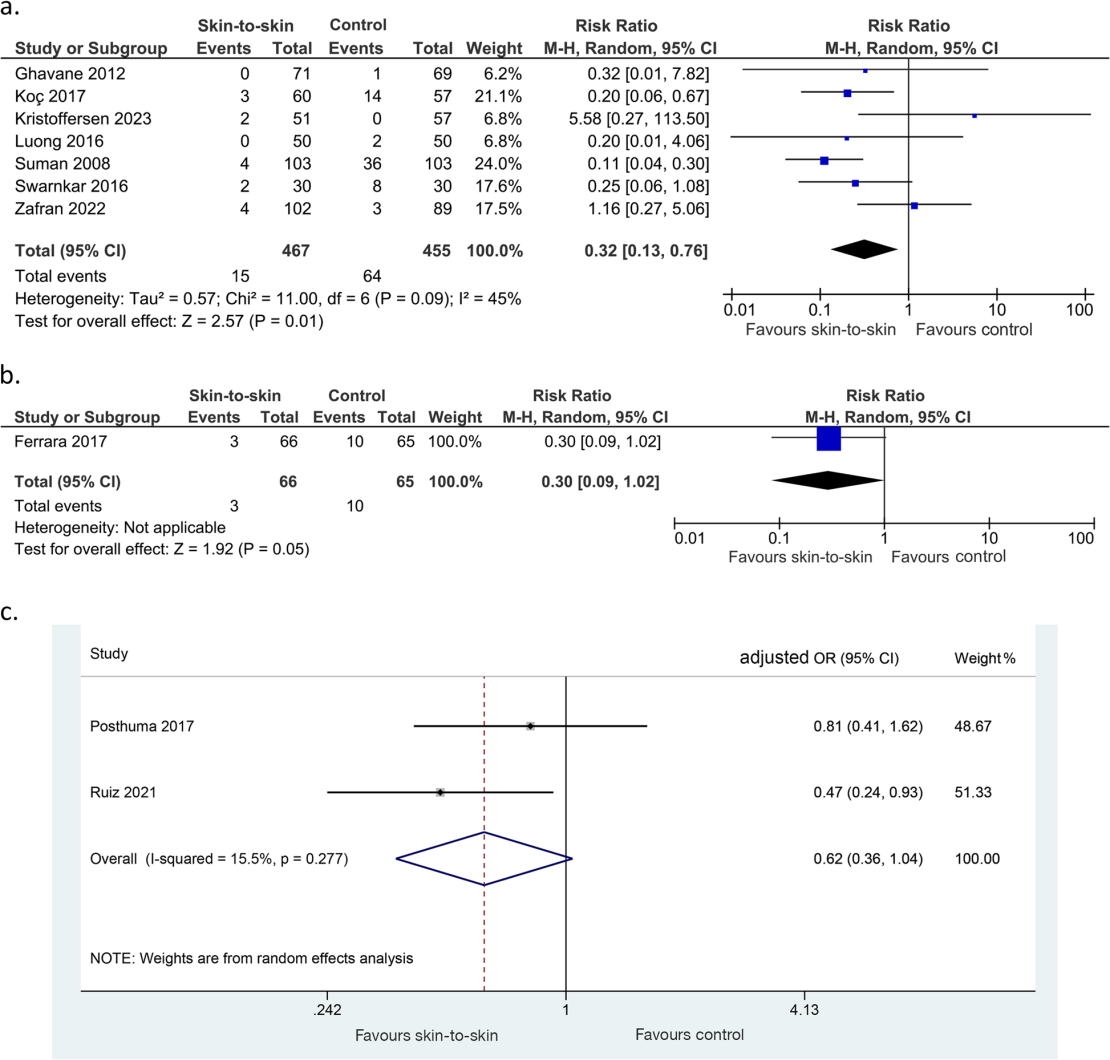
Effect of skin-to-skin contact on neonatal hypoglycaemia. **a** Results from randomised or quasi-randomised controlled trials. **b** Results from non-randomised studies of interventions. **c** Results from cohort studies

Evidence from seven RCTs or quasi-RCTs showed that skin-to-skin contact may result in a large reduction in the incidence of neonatal hypoglycaemia (922 infants, RR 0.32 (0.13, 0.76), *p* = 0.01, I^2^ = 45%, low certainty of evidence, Fig. 3a). Evidence from one non-randomised study of intervention also suggested a reduction in neonatal hypoglycaemia (131 infants, RR 0.30 (0.09, 1.02), *p* = 0.05, Fig. 3b). Evidence from two cohort studies was very uncertain about the effect of skin-to-skin on neonatal hypoglycaemia (863 infants, adjusted OR 0.62 (0.36, 1.04), *p* = 0.07, I^2^ = 15.5%, Fig. 3c). Linner 2022 [31], an RCT, found that neonatal hypoglycaemia incidence was similar between the intervention and control groups but did not provide raw data (101 infants).

### Secondary outcomes:

#### Admission to special care nursery or neonatal intensive care nursery

**Fig. 4:**
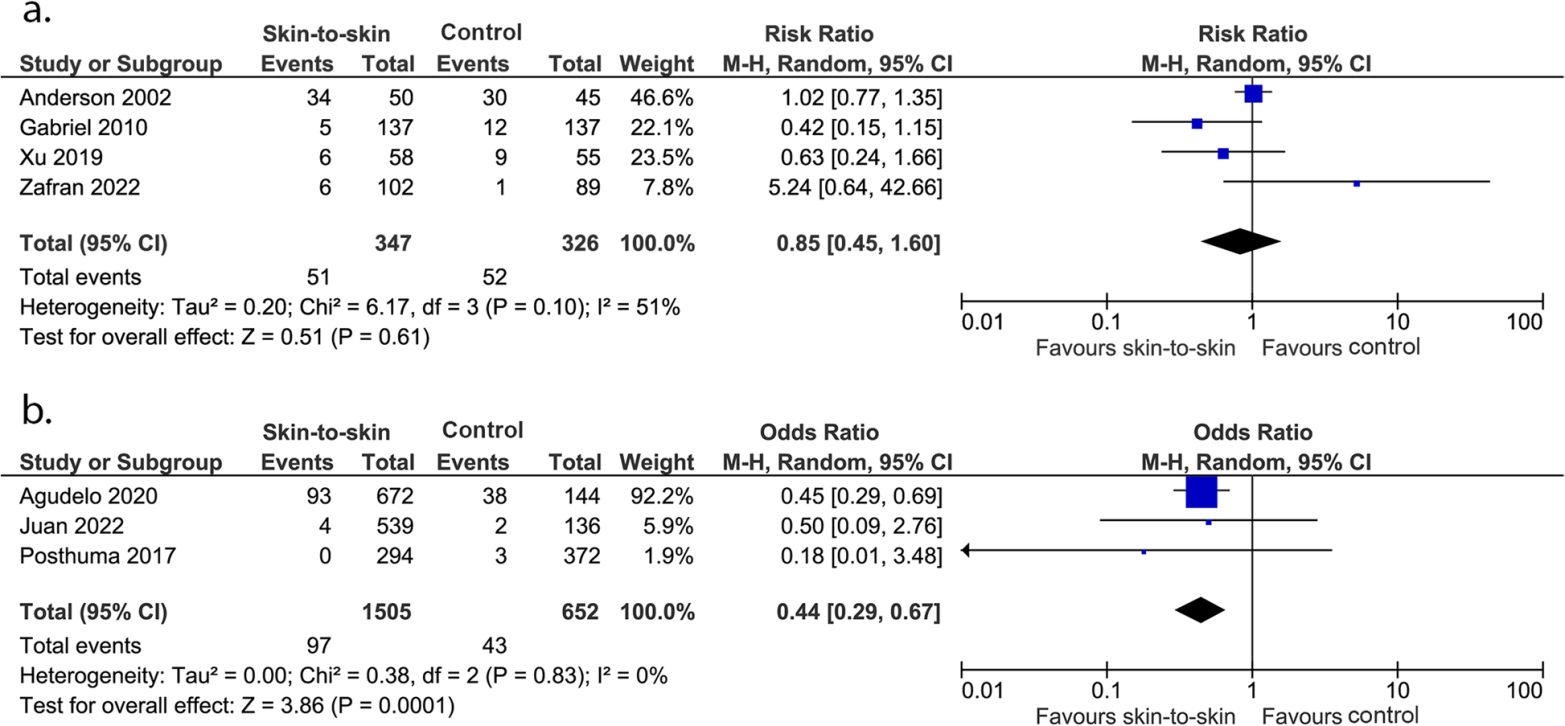
Effect of skin-to-skin contact on admission rate to special care or neonatal intensive care nursery. **a** Results from randomised or quasi-randomised controlled trials. **b** Results from cohort studies

Evidence from four RCTs showed that skin-to-skin contact has little to no effect on admission to a special care or neonatal intensive care nursery (673 infants, RR 0.85 (0.45, 1.60), *p* = 0.61, I^2^ = 51%, Fig. 4a). However, very uncertain evidence from three cohort studies showed that skin-to-skin contact may reduce admission to a special care or neonatal intensive care nursery (2157 infants, OR 0.44 (0.29, 0.67), *p* = 0.0001, I^2^ = 0%, Fig. 4b).

#### Special care or neonatal intensive care nursery admission for hypoglycaemia

**Fig. 5:**
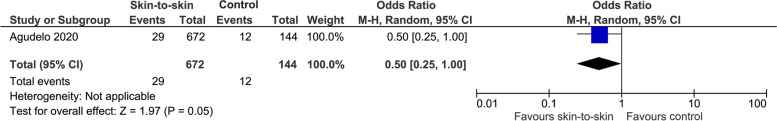
Effect of skin-to-skin contact on admission to neonatal special or intensive care nursery for hypoglycaemia

Skin-to-skin contact may reduce special care or neonatal intensive care nursery admission for hypoglycaemia, but the evidence is very uncertain (1 cohort study, 816 infants, OR 0.50 (0.25, 1.00), *p* = 0.05, Fig. 5).

#### Hypothermia (study defined)

**Fig. 6:**
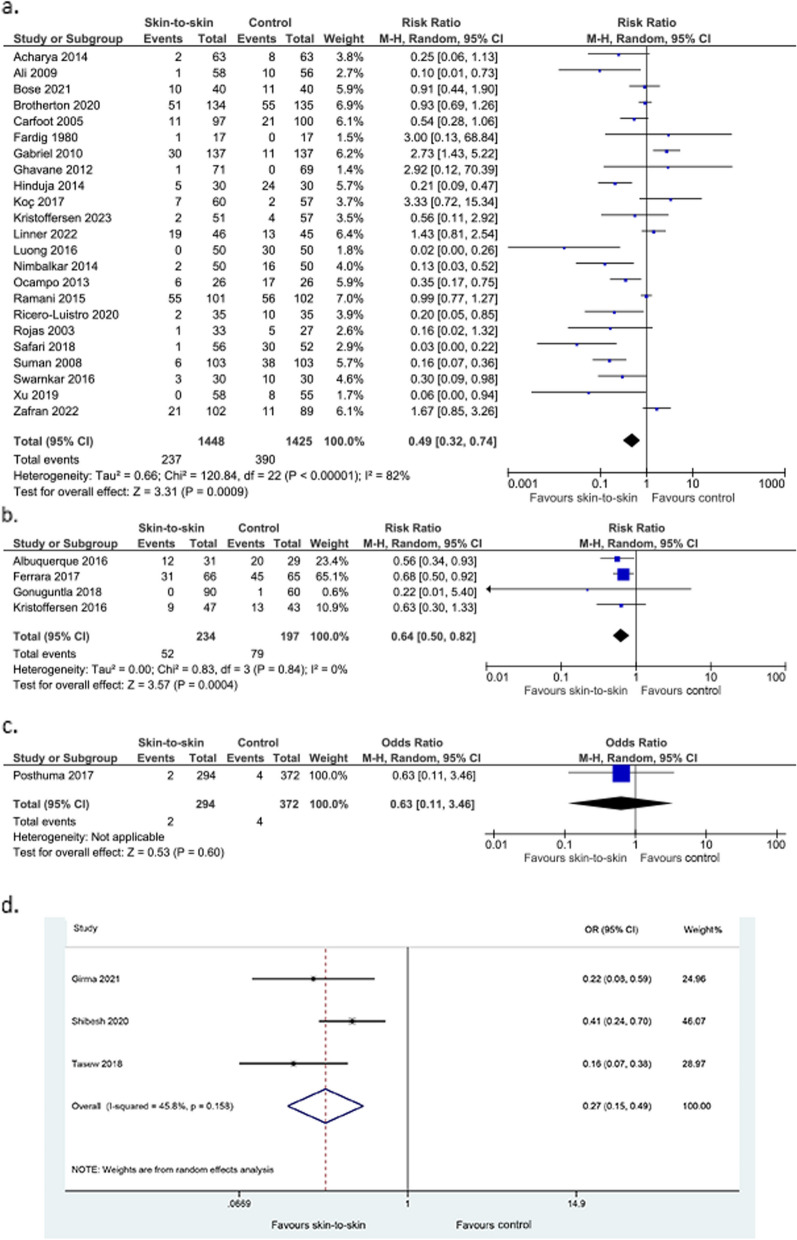
Effect of skin-to-skin contact on hypothermia. **a** Results from randomised or quasi-randomised controlled trials **b** Results from non-randomised studies of intervention **c** Results from cohort studies. **d** Results from case–control studies

Skin-to-skin contact may lead to a large reduction in the incidence of hypothermia but the evidence is very uncertain, with high heterogeneity and significant publication bias (23 RCTs, 2873 infants, RR 0.49 (0.32, 0.74), *p* = 0.0009, I^2^ = 82%, Fig. 6a; p for Egger’s test = 0.03; 4 non-randomised studies of intervention, 431 infants, RR 0.64 (0.50, 0.82) *p* = 0.0004, I^2^ = 0%, Fig. 6b; 1 cohort study, 666 infants, OR 0.63 (0.11–3.46), *p* = 0.60, Fig. 6c; 3 case–control studies, 870 infants, adjusted OR 0.27 (0.15, 0.49), *p* < 0.0001, I^2^ = 45.8%, Fig. 6d). Kanodia 2016 [32], an RCT, found a reduced rate of hypothermia in the skin-to-skin group; 5.1% compared to 14.6% in the control group (242 infants, *p* = 0.048). They did not provide raw data, so this has not been included in the meta-analysis. Kadam 2005 [33], another RCT, found a reduction in episodes of hypothermia in the intervention group compared to the control group (10/44, 23%, versus 21/45, 47%, *p* < 0.01). This data was not included in the meta-analysis as it reported episodes rather than the incidence of hypothermia.

#### Hyperthermia (study defined)

**Fig. 7:**
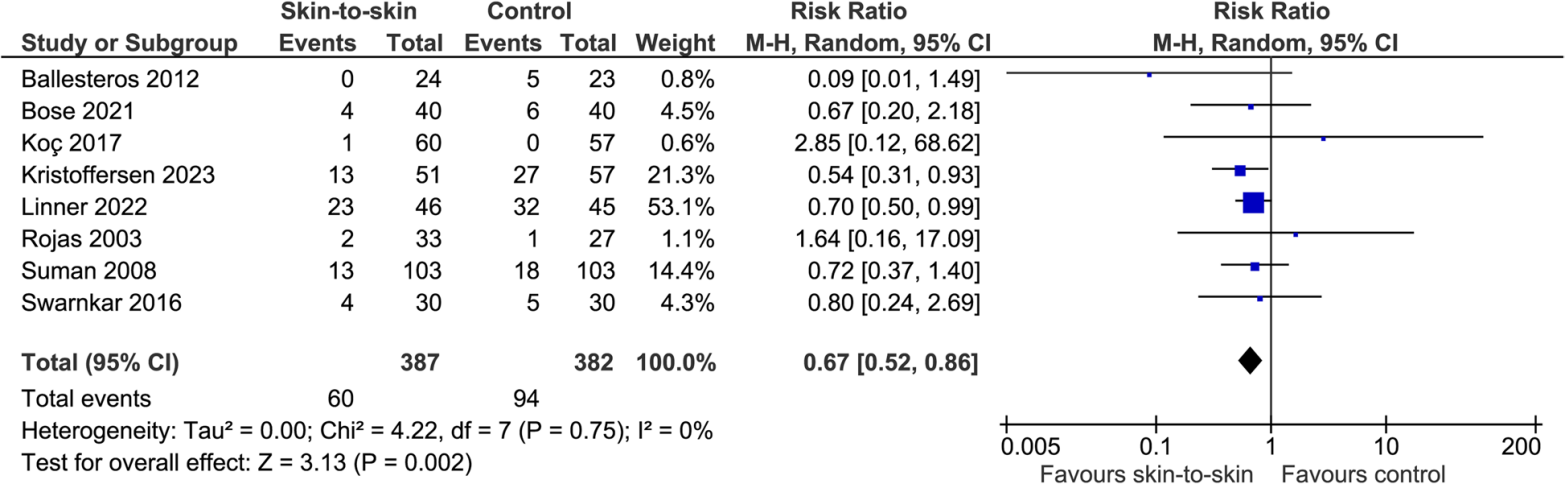
Effect of skin-to-skin contact on hyperthermia

The evidence suggests skin-to-skin contact results in a reduction in the incidence of hyperthermia (8 RCTs, 769 infants, RR 0.67 (0.52, 0.86), *p* = 0.002, I^2^ = 0%, Fig. 7). Kadam 2005 [33], another RCT, found no statistically significant difference in episodes of hyperthermia between the intervention and control group (13/44, 30%, versus 15/45, 33%).

#### Adverse effects (study defined)

Rojas 2003 [34] found that the frequency of adverse events, including apnoea, desaturations and regurgitations, was not increased in the skin-to-skin contact group (60 infants). Similarly, Linner 2022 [31] found no difference in the frequency or severity of adverse events between the intervention and control groups (91 infants). Neither study reported the total number of infants in each group experiencing adverse events so meta-analysis was not possible.

#### Duration of initial hospital stay after birth

**Fig. 8:**
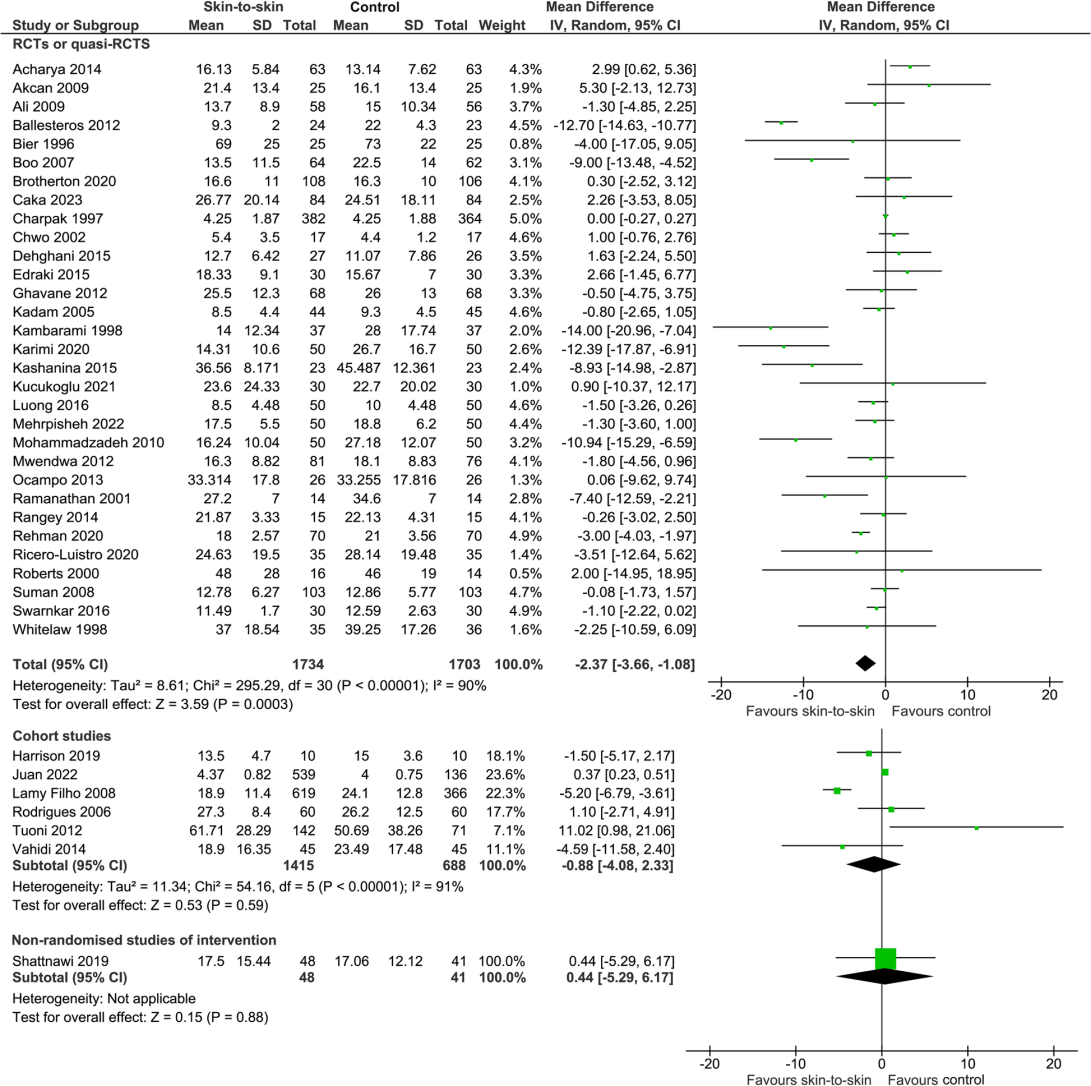
Effect of skin-to-skin contact on duration of hospital stay (days)

Skin-to-skin contact may reduce the duration of initial hospital stay after birth but the evidence is very uncertain with significant publication bias (31 RCTs, 3437 infants, MD -2.37 (-3.66, -1.08) days, *p* = 0.0003, I^2^ = 90%, p for Egger’s test = 0.02; 6 cohort studies, 2103 infants, MD -0.88 (-4.08, 2.33) days, *p* = 0.59, I^2^ = 91%; 1 non-randomised study of intervention, 89 infants, MD 0.44 (-5.29, 6.17) days, *p* = 0.88) (Fig. 8). Sloan 1994 [35] showed in an RCT that infants who received skin-to-skin contact stayed in the hospital for an average of 2 days longer than those who received standard care, but raw data were not provided. However, this may have been because the infants who received skin-to-skin contact on average had a gestational age that was 15 days earlier than those in the standard care group. Kanodia 2016 [32] also found that duration of initial hospital stay after birth was longer for infants who received KMC but raw data were not provided (242 infants). In contrast, Worku 2005 [36] found that infants in the KMC group were discharged at a mean age of 4.4 days, compared to the mean age of 5.4 days in the comparison group. They stated this difference was statistically significant. However, the standard deviation and number of infants in each group were not reported so this data could not be included in the meta-analysis.

#### Exclusive breastmilk feeding from birth to discharge

**Fig. 9:**
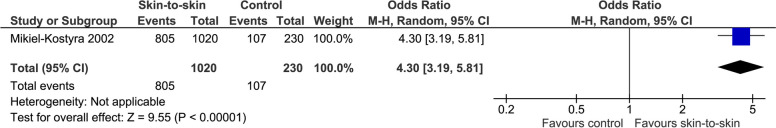
Effect of skin-to-skin contact on exclusive breastmilk feeding from birth to discharge

The evidence suggests skin-to-skin contact may result in a large increase in the rate of exclusive breastmilk feeding from birth to discharge but the evidence is very uncertain (1 observational study, 1250 infants, OR 4.30 (3.19, 5.81), *p* < 0.0001, Fig. 9).

#### Exclusive breastmilk feeding at discharge

**Fig. 10:**
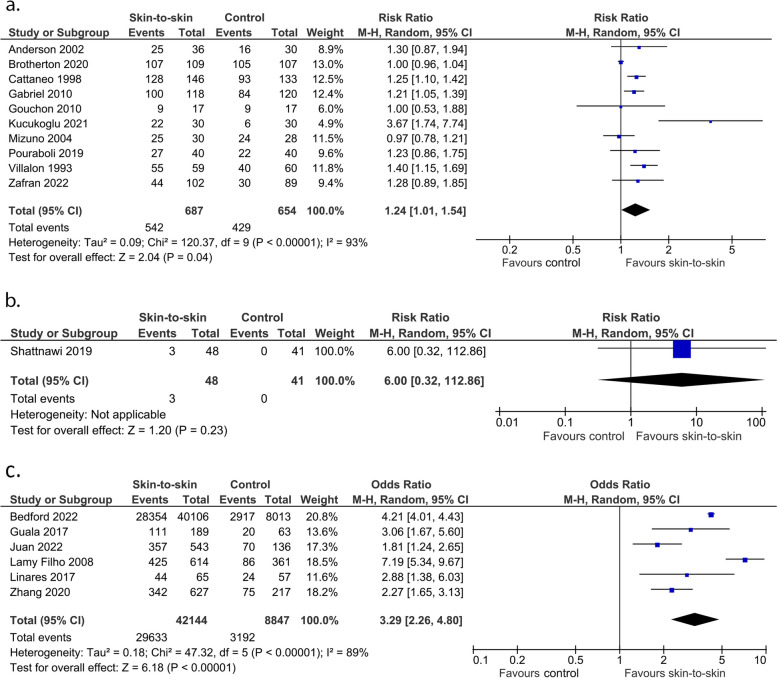
Effect of skin-to-skin contact on exclusive breastmilk feeding at discharge. **a** Results from randomised or quasi-randomised controlled trials. **b** Results from non-randomised studies of intervention **c** Results from cohort studies

The evidence suggests skin-to-skin contact may increase the rate of exclusive breastmilk feeding at discharge (10 RCTs, 1341 infants, RR 1.24 (1.01, 1.54), *p* = 0.04, I^2^ = 93%, p for Egger’s test = 0.02, Fig. 10a; 1 non-randomised study of intervention, 89 infants, RR 6.00 (0.32, 112.86), *p* = 0.23, Fig. 10b; 6 cohort studies, 50,991 infants, OR 3.29(2.26, 4.80), *p* < 0.0001, I^2^ = 89%, Fig. 10c), however the evidence is very uncertain with substantial heterogeneity. Sheedy 2022 [37], a retrospective cohort study, found that skin-to-skin contact increased the rate of exclusive breastfeeding at discharge (OR 2.24; 95% CI 1.79–2.82) but did not provide the raw data.

#### Exclusive breastmilk feeding at term equivalent age: not pre-specified

**Fig. 11:**
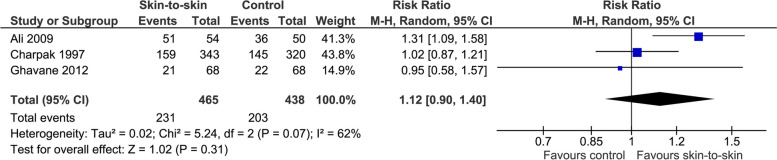
Effect of skin-to-skin contact on exclusive breastmilk feeding at term equivalent age

The effect of skin-to-skin contact on exclusive breastmilk feeding at term equivalent age (not a pre-specified outcome) is very uncertain (3 RCTs, 903 infants, RR 1.12 (0.90, 1.40), *p* = 0.31, I^2^ = 62%, Fig. 11).

#### Exclusive breastmilk feeding within the period from discharge to 3 months: not- pre-specified

**Fig. 12:**
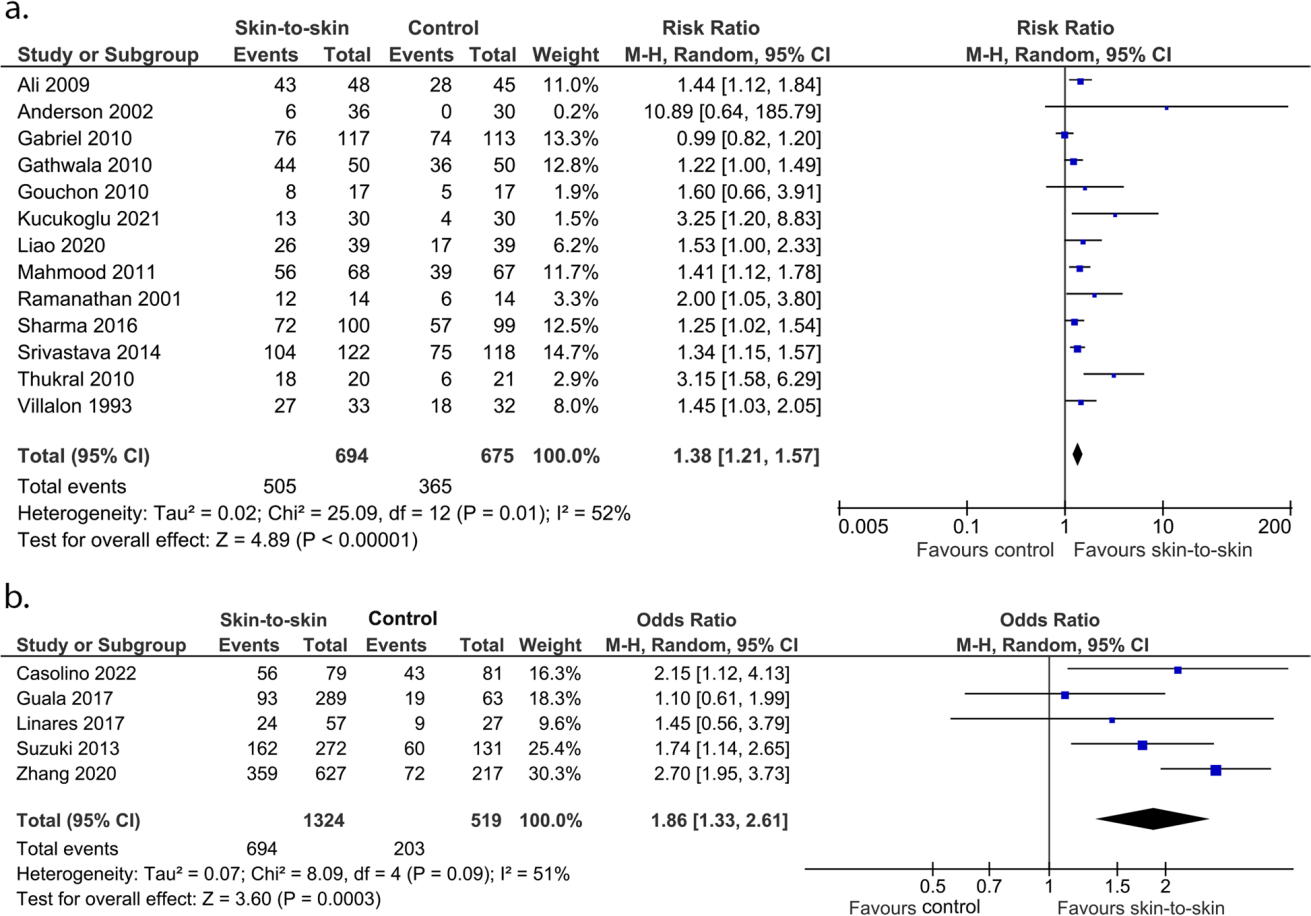
Effect of skin-to-skin contact on exclusive breastmilk feeding within period from discharge to 3 months. **a** Results from randomised or quasi-randomised controlled trials. **b** Results from cohort studies. The results from some studies are reported at corrected age and some are at postnatal age

Skin-to-skin contact may increase the rate of exclusive breastmilk feeding from discharge to 3 months (not a pre-specified outcome) but the evidence is very uncertain with substantial heterogeneity and significant publication bias (13 RCTs, 1369 infants, RR 1.38 (1.21, 1.57), *p* < 0.0001, I^2^ = 52%, p for Egger’s test = 0.002, Fig. 12a; 5 cohort studies, 1843 infants, OR 1.86 (1.33, 2.61), p = 0.0003), I^2^ = 40%, Fig. 12b).

#### Exclusive breastmilk feeding within the period from 3 to 6 months: not pre-specified

**Fig. 13:**
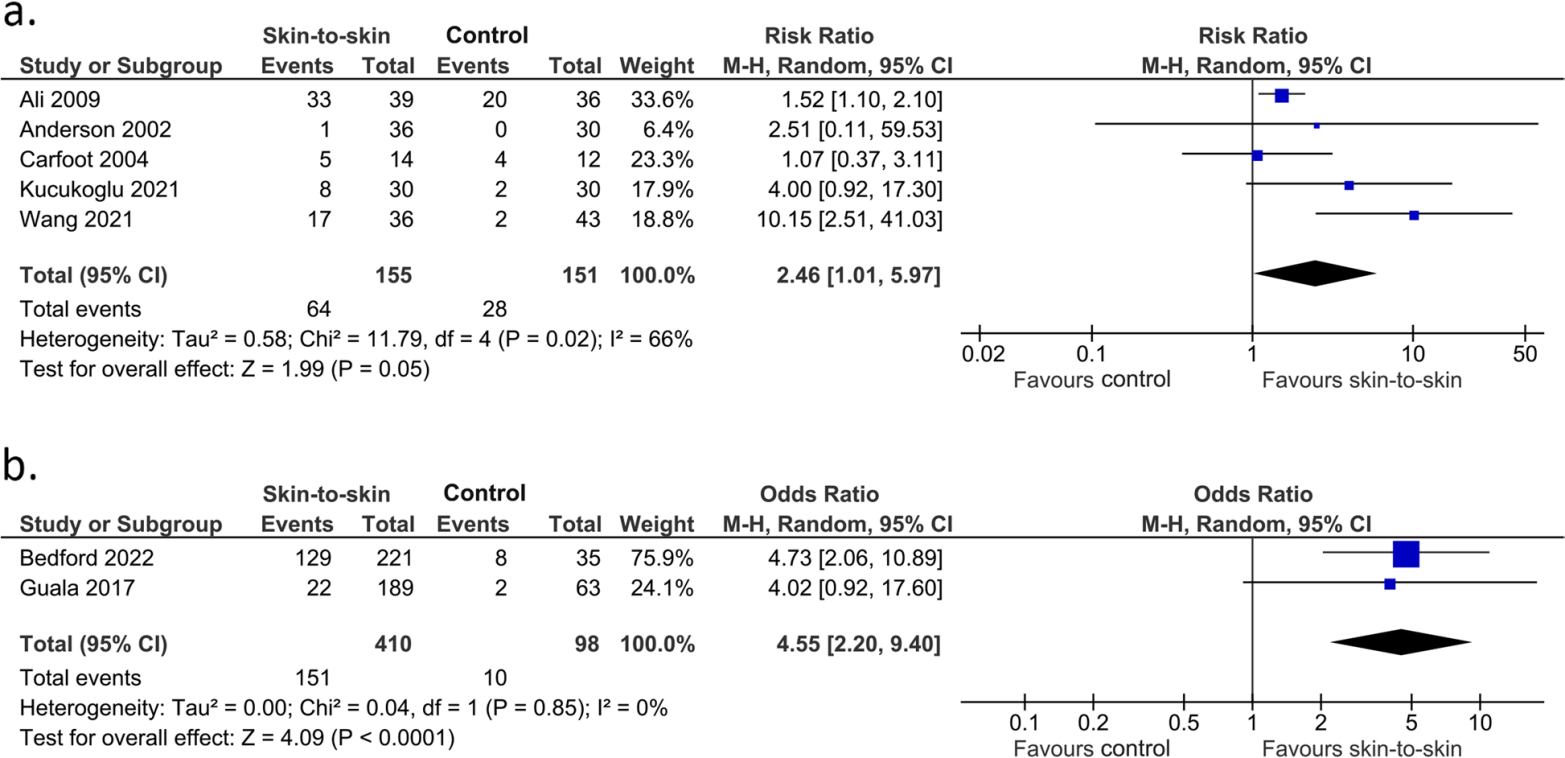
Effect of skin-to-skin contact on exclusive breastmilk feeding within the period from 3 to 6 months. **a** Results from randomised or quasi-randomised controlled trials. **b** Results from cohort studies. The results from some studies are reported at corrected age and some are at postnatal age

Skin-to-skin contact may increase the rate of exclusive breastmilk feeding from 3 to 6 months (not a pre-specified outcome) (5 RCTs, 306 infants, RR 2.46 (1.01, 5.97), *p* = 0.05, I^2^ = 66%, Fig. 13a; 2 cohort studies, 508 infants, OR 4.55 (2.20, 9.40), *p* < 0.0001, I^2^ = 0%, Fig. 13b).

#### Any breastmilk feeding (not pre-specified)

**Fig. 14:**
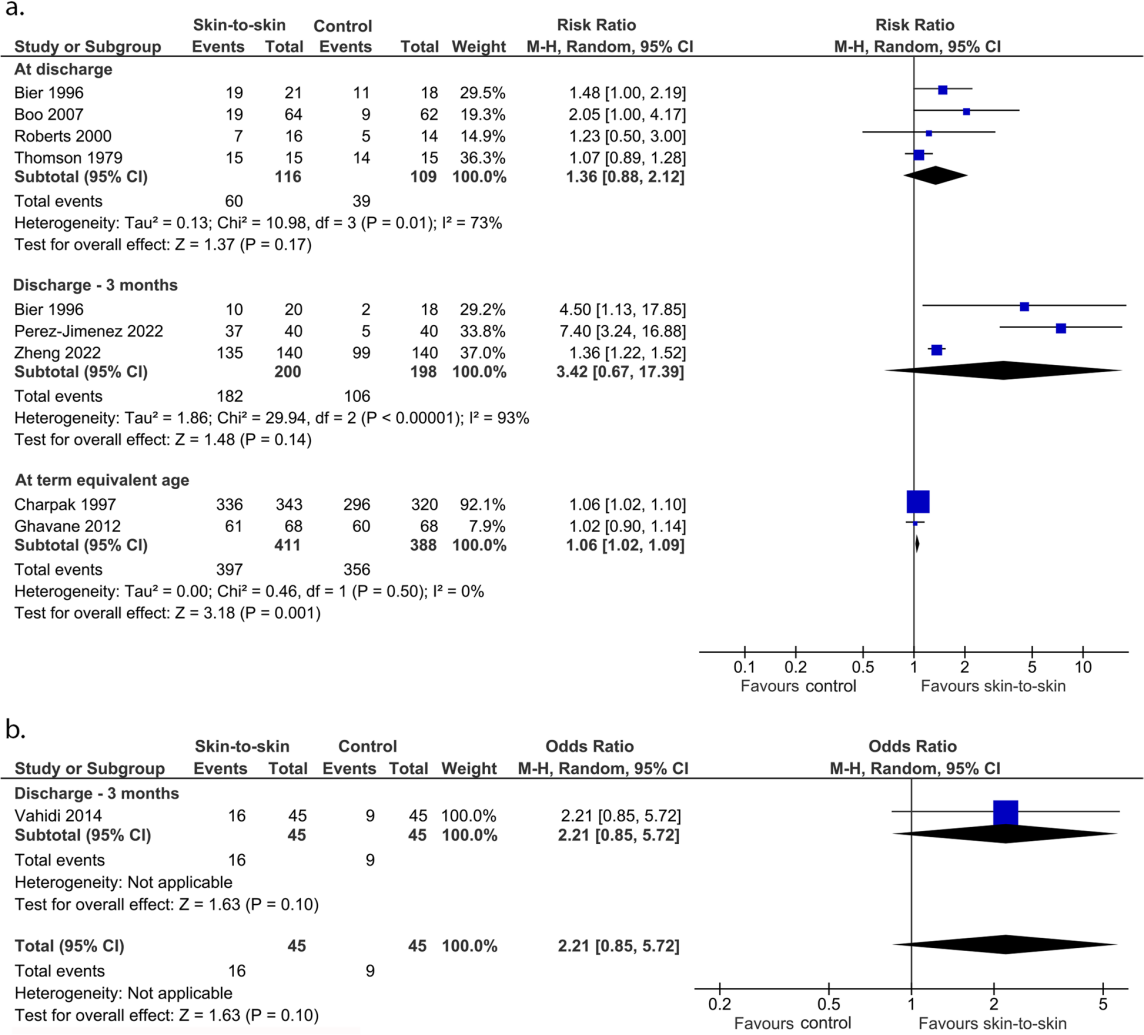
Effect of skin-to-skin contact on any breastmilk feeding. **a** Results from randomised or quasi-randomised controlled trials. **b** Results from cohort studies

These outcomes were not pre-specified. It is very uncertain whether skin-to-skin contact affects the rate of any breastmilk feeding at discharge (4 RCTs, 225 infants, RR 1.36 (0.88, 2.12), *p* = 0.17, I^2^ = 73%, Fig. 14a; 1 cohort study, 90 infants, OR 2.21 (0.85, 5.72), *p* = 0.10, Fig. 14b) or within the period from discharge to 3 months (3 RCTs, 398 infants, RR 3.42 (0.67, 17.39), *p* = 0.14, I^2^ = 93%, Fig. 14a). Charpak 1997 [38] found 81.7% of infants in the KMC group and 75.3% of infants in the control group consumed any breastmilk at 3 months corrected age (*p* = 0.05). They found no statistically significant difference between the rate of any breastfeeding at 6, 9 and 12 months corrected age. These findings could not be included in the meta-analysis as no raw data were provided. Skin-to-skin contact likely results in a slight increase in any breastmilk feeding at term equivalent age (2 RCTs, 799 infants, RR = 1.06 (1.02, 1.09), *p* = 0.001, I^2^ = 0%, Fig. 14a). For this analysis, we included data provided in Bier 1996 from the time most similar to the other studies rather than the latest time.

#### Blood glucose concentrations

**Fig. 15:**
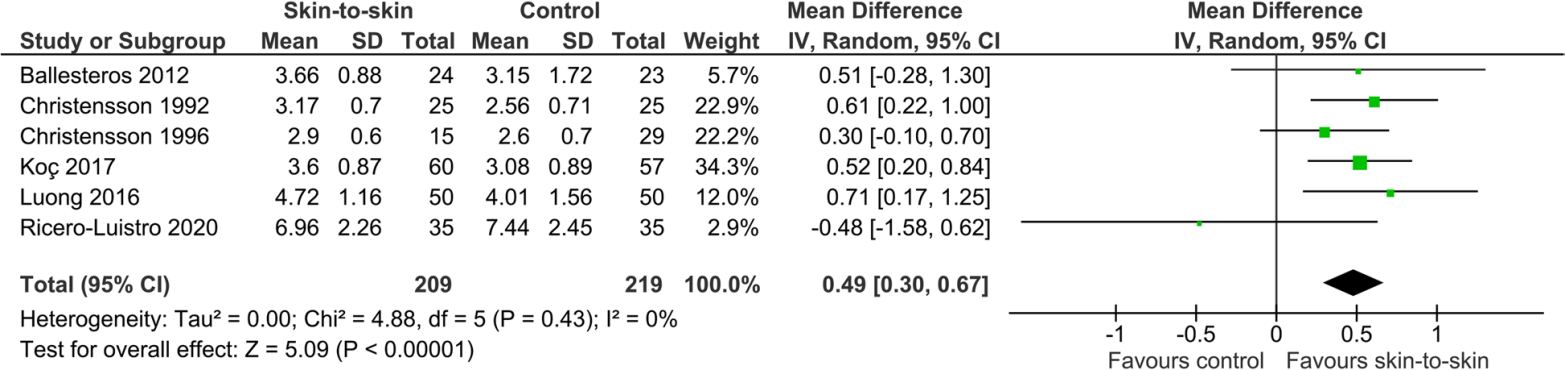
Effect of skin-to-skin contact on blood glucose concentration (mmol/l)

Six RCTs including 428 infants investigated the effect of skin-to-skin contact on blood glucose concentrations, but they measured these at different times. Nonetheless, the evidence suggests that skin-to-skin contact increases blood glucose concentration, with a mean difference of 0.49 mmol/l (0.30–0.67), I^2^ = 0%, *p* < 0.0001, Fig. 15). Suciu 2016 [39] found in an RCT that the blood glucose concentrations of infants who received skin-to-skin contact was 1.14 mmol/l higher than those who received standard care 75 to 90 min after birth (64 infants), but raw data were not provided.

#### Temperature

Thirty-seven studies, consisting of 34 RCTs or quasi-RCTs and 3 non-randomised studies of intervention, investigated the effects of skin-to-skin contact on infant temperature. However, these were measured in different ways and at different times, making it difficult to conduct a meta-analysis. Of the 37 studies, 18 reported no differences in temperature between the skin-to-skin contact and the standard care groups during or after the intervention. Eighteen studies reported that infants who received skin-to-skin contact had higher temperatures, and one study reported that infants who received skin-to-skin contact had lower temperatures than those who received standard care (Table 1).

**Table 1:** Effect of skin-to-skin contact on infant body temperature

Higher temperature in the skin-to -skin group	Similar temperature between groups	Lower temperature in the skin-to-skin group
Albuquerque 2016	Ayala 2021	Toprak 2022
Çaka 2023	Beiranvand 2014	
Carfoot 2005	Bier 1996	
Christensson 1992	Bose 2021	
Christensson 1996	Britton 1980	
Chwo 2002	Bystrova 2003	
Dehghani 2015	Gouchon 2010	
Gathwala 2010	Kristoffersen 2023	
Hinduja 2014	Ludington-Hoe 2004	
Keshavarz 2010	Myron 2017	
Koç 2017	Olmedo 2012	
Li 2022	Parsa 2018	
Ludington-Hoe 2000	Roberts 2000	
Mathews 2018	Sharma 2016	
Nimbalkar 2014	Solanki 2021	
Rangey 2014	Srivastava 2014	
Safari 2018	Villalon 1993	
Xu 2019	Yin 2000	

#### Other outcomes (not pre-specified)

Charpak 1997 [38], an RCT, found no overall differences in mean intelligence scores at 20 years between the adults who received skin-to-skin contact during the neonatal period and those who received standard care (139 participants, mean score 87.5 ± 13.8 vs 125 participants, 88.4 ± 13.9). However, a subgroup of 63 children who were identified as neurologically vulnerable (determined by neurologic examination, no details provided) at 6 months of age showed higher scores in intelligence and attention in adulthood if they had received skin-to-skin contact during the neonatal period. Moreover, young adults who had received skin-to-skin contact during the neonatal period had larger volumes of brain structures associated with intelligence, attention, memory, and coordination compared to those who received standard care (178 participants). [38]. Harrison 2019 [40] found that neonatal skin-to-skin contact could improve learning and autonomic development in 3-month-old infants with complex congenital heart disease (20 participants). They reported increased engagement with a learning task (reduced parasympathetic activation), improved heart rate variability regulation during the task and greater recovery afterwards (reduced heart rate).

### Subgroup analyses

There was a significant interaction between the duration of skin-to-skin contact and the incidence of hypothermia, whereby infants who received skin-to-skin contact had a lower incidence of hypothermia than infants who did not if the skin-to-skin contact lasted ≥ 60 min, but not if it lasted < 60 min (*p* = 0.0005 for interaction). There was a significant interaction between timing of skin-to-skin contact and hypothermia, with skin-to-skin contact initiated at least 24 hours after birth reducing the incidence of hypothermia, but not if it was initiated within 24 hours after birth. There was a significant interaction between timing of skin-to-skin contact and blood glucose concentration, with infants who received skin-to-skin contact initiated within the first 10 min after birth having an increased blood glucose concentration compared to infants who did not, but not if the skin-to-skin contact was initiated after 24 h (*p* = 0.03 for interaction). There were significant interactions between preterm versus term infants and the impact of skin-to-skin contact on exclusive breastmilk feeding from discharge to 3 months and any breastmilk feeding after discharge. Preterm infants experienced a greater benefit of skin-to-skin contact on exclusive breastmilk feeding from discharge to 3 months (*p* = 0.03 for interaction) and term infants experienced a greater benefit of skin-to-skin contact on any breastmilk feeding after discharge (*p* = 0.04 for interaction).

There was a significant interaction between mode of delivery and whether skin-to-skin contact reduced the incidence of neonatal hypoglycaemia, with infants delivered vaginally who received skin-to-skin contact having lower rates of neonatal hypoglycaemia than the control group, but no difference between intervention and control groups for infants delivered by Caesarean section (*p* = 0.02 for interaction). In addition, infants delivered vaginally who received skin-to-skin contact had higher blood glucose concentrations than the control group, but there was no difference between intervention and control group for infants delivered by Caesarean section (*p* = 0.02 for interaction). Due to insufficient data, we were unable to undertake other preplanned subgroup analyses of infants at risk of neonatal hypoglycemia versus not at risk, single versus multiple births, and skin-to-skin contact with mother versus skin-to-skin contact with another person (Table 2). We were also unable to conduct sensitivity analyses for outcomes with significant heterogeneity by excluding studies at high risk of bias, as we only judged one study to be high quality.

**Table 2:** Summary of subgroup analysis

Outcomes	Subgroups	No. of participants (studies)	Relative risk (RR) or mean difference (MD) (95% CI)	*P* for overall effect	I^2^	*P* for subgroup interaction
**Duration of skin-to-skin contact**						
Neonatal hypoglycaemia	< 60 min	308 (2)	0.46 (0.08, 2.55)	0.37	70%	0.68
	≥ 60 min	514 (4)	0.29 (0.08, 1.10)	0.07	52%	
Admission to special care nursery or neonatal intensive care	< 60 min	191 (1)	5.24 (0.64, 42.66)	0.12	N/A	0.08
	≥ 60 min	482 (3)	0.74 (0.45, 1.60)	0.34	54%	
Hypothermia	< 60 min	625 (5)	1.16 (0.82, 1.64)	0.39	22%	0.0005
	≥ 60 min	1951 (16)	0.34 (0.18, 0.62)	0.0003	83%	
Hyperthermia	< 60 min	197 (2)	0.80 (0.26, 2.42)	0.69	0%	0.76
	≥ 60 min	572 (6)	0.66 (0.51, 0.86)	0.002	0%	
Exclusive breastmilk feeding at discharge	< 60 min	271 (2)	1.25 [0.97, 1.62]	0.08	0%	0.95
	≥ 60 min	893 (6)	1.27 [0.94, 1.71]	0.12	95%	
Exclusive breastmilk feeding within the period from discharge to 3 months	< 60 min	199 (1)	1.25 [1.02, 1.54]	0.04	N/A	0.32
	≥ 60 min	970 (10)	1.44 [1.19, 1.74]	0.0001	63%	
Any breastmilk feeding at discharge	< 60 min	29 (1)	1.07 [0.89, 1.28]	0.47	N/A	0.07
	≥ 60 min	156 (2)	1.24 [0.43, 3.55]	0.07	84%	
Blood glucose concentration	< 60 min	117 (1)	0.52 [0.20, 0.84]	0.001	N/A	0.33
	≥ 60 min	211 (4)	0.25 [-0.18, 0.69]	0.26	56%	
**Timing of initiation of skin-to-skin contact**						
Neonatal hypoglycaemia	Immediate ≤ 10 min	408 (3)	0.40 (0.11, 1.43)	0.16	42%	0.05
	Early > 10 min to 24 h	108 (1)	5.58 (0.27 – 113.50)	0.26	N/A	
	> 24 h to discharge	406 (3)	0.15 (0.07, 0.34)	< 0.0001	0%	
Hypothermia	Immediate ≤ 10 min	1488 (11)	0.67 (0.35, 1.31)	0.25	85%	0.02
	Early > 10 min to 24 h	557 (4)	0.63 (0.31, 1.28)	0.20	64%	
	> 24 h to discharge	588 (6)	0.25 (0.16, 0.40)	< 0.0001	0%	
Hyperthermia	Immediate ≤ 10 min	208 (2)	0.71 (0.51, 1.01)	0.05	0%	0.70
	Early > 10 min to 24 h	188 (2)	0.56 (0.34, 0.92)	0.02	0%	
	> 24 h to discharge	373 (4)	0.71 (0.41, 1.24)	0.23	0%	
Duration of hospital stays (days)	Immediate ≤ 10 min	100 (1)	-1.50 (-3.26, 0.26)	0.09	N/A	0.20
	Early > 10 min to 24 h	301 (2)	0.19 (-2.56, 2.94)	0.89	0%	
	> 24 h to discharge	2386 (19)	-2.70 [-4.36, -1.03]	0.002	93%	
Exclusive breastmilk feeding at discharge	Immediate ≤ 10 min	553 (4)	1.15 (1.02, 1.30)	0.02	12%	0.62
	Early > 10 min to 24 h	449 (4)	1.16 (078, 1.72)	0.48	93%	
	> 24 h to discharge	279 (1)	1.25 [1.10, 1.42]	0.0005	N/A	
Exclusive breastmilk feeding within the period from discharge to 3 months	Immediate ≤ 10 min	671 (5)	1.21 [0.98, 1.49]	0.07	52%	0.43
	Early > 10 min to 24 h	417 (4)	1.38 [1.21, 1.58]	< 0.0001	0%	
	> 24 h to discharge	823 (3)	1.23 [1.04, 1.45]	0.01	0%	
Exclusive breastmilk feeding within the period from 3 to 6 months	Immediate ≤ 10 min	66 (1)	2.51 [0.11, 59.53]	0.57	N/A	0.62
	Early > 10 min to 24 h	26 (1)	1.07 [0.37, 3.11]	0.90	N/A	
	> 24 h to discharge	0	-	-	-	
Any breastmilk feeding at discharge	Immediate ≤ 10 min	0	-	-	-	0.77
	Early > 10 min to 24 h	29 (1)	1.07 [0.89, 1.28]	0.47	N/A	
	> 24 h to discharge	30 (1)	1.23 [0.50, 3.00]	0.66	N/A	
Blood glucose concentration	Immediate ≤ 10 min	267 (3)	0.58 [0.36, 0.81]	< 0.0001	0%	0.03
	Early > 10 min to 24 h	44 (1)	0.00 [-0.40, 0.40]	1.00	N/A	
	> 24 h to discharge	117 (2)	0.09 [-0.86, 1.05]	0.85	51%	
**Babies born preterm versus at term**						
Neonatal hypoglycaemia	Preterm	248 (2)	1.41 (0.09, 22.99)	0.81	38%	0.90
	Term	191 (1)	1.16 (0.27, 5.06)	0.84	N/A	
Admission to special care nursery or neonatal intensive care	Preterm	208 (2)	0.98 (0.74, 1.30)	0.88	2%	0.12
	Term	191 (1)	5.24 (0.64, 42.66)	0.12	N/A	
Hypothermia	Preterm	911 (8)	0.57 (0.29, 1.11)	0.10	64%	0.68
	Term	590 (5)	0.43 (0.13, 1.43)	0.17	79%	
Exclusive breastmilk feeding at discharge	Preterm	126 (2)	2.01 [0.62, 6.58]	0.25	88%	0.39
	Term	482 (5)	1.19 [1.00, 1.41]	0.05	39%	
Exclusive breastmilk feeding within the period from discharge to 3 months	Preterm	154 (3)	2.42 [1.44, 4.08]	0.0009	0%	0.03
	Term	781 (7)	1.36 (1.23, 1.51)	< 0.0001	0%	
Any breastmilk feeding at discharge	Preterm	69 (2)	1.44 [1.00, 2.06]	0.05	0%	0.15
	Term	30 (1)	1.07 [0.89, 1.28]	0.47	N/A	
Any breastmilk feeding within the period from discharge to 3 months	Preterm	38 (1)	4.50 [1.13, 17.85]	0.03	N/A	0.04
	Term	80 (1)	7.40 [3.24, 16.88]	< 0.0001	N/A	
Blood glucose concentration	Preterm	70 (1)	-0.48 [-1.58, 0.62]	0.39	N/A	0.22
	Term	94 (2)	0.31 [-0.29, 0.90]	0.32	78%	
**Vaginal birth versus caesarean birth**						
Neonatal hypoglycaemia	Vaginal birth	163 (2)	0.20 (0.06, 0.67)^a^	0.009	N/A	0.02
	Caesarean section	253 (2)	1.56 (0.42, 5.85)	0.51	0%	
Admission to special care nursery or neonatal intensive care	Vaginal birth	274 (1)	0.42 (0.15, 1.15)	0.09	N/A	0.28
	Caesarean section	304 (2)	1.49 (0.18, 12.04)	0.71	71%	
Hypothermia	Vaginal birth	1056 (9)	0.48 (0.19, 1.24)	0.13	86%	0.34
	Caesarean section	446 (4)	0.89 (0.38, 2.09)	0.79	81%	
Hyperthermia	Vaginal birth	163 (2)	0.78 (0.41, 1.48)	0.52	0%	0.58
	Caesarean section	142 (2)	0.47 (0.23, 0.95)	0.04	0%	
Exclusive breastmilk feeding at discharge	Vaginal birth	415 (3)	1.19 [0.99, 1.43]	0.06	67%	0.90
	Caesarean section	305 (3)	1.21 (0.96, 1.54)	0.11	0%	
Exclusive breastmilk feeding within the period from discharge to 3 months	Vaginal birth	1081 (10)	1.35 [1.17, 1.55]	< 0.0001	58%	0.71
	Caesarean section	34 (1)	1.60 (0.66, 3.91)	0.30	N/A	
Blood glucose concentration	Vaginal birth	167 (2)	0.56 [0.31, 0.80]	< 0.0001	0%	0.02
	Caesarean section	44 (1)	0.00 [-0.40, 0.40]	1.00	N/A	

^a^RR not estimable from Kristofferson 2023 due to 0 events in most groups

### Certainty of Evidence (GRADE assessment)

The certainty of the evidence was assessed as low for neonatal hypoglycaemia and very low for special care nursery or neonatal intensive care nursery admission for hypoglycaemia, duration of initial hospital stay after birth and exclusive breastmilk feeding from birth to discharge. There were no data for the outcomes: receipt of treatment for hypoglycaemia and hypoglycaemic injury on brain imaging (Table 3).

**Table 3:** GRADE assessment

Outcomes	№ of participants (studies) Follow-up	Certainty of the evidence (GRADE)	Relative effect (95% CI)	Anticipated absolute effects	
				**Risk with standard care**	**Risk difference with skin-to-skin contact**
Neonatal hypoglycaemia (study-defined)	922 (7 RCTs)	⨁⨁◯◯ Low^a^	**RR 0.32** (0.13 to 0.76)	163 per 1,000	**111 fewer per 1,000** (141 fewer to 39 fewer)
Special care nursery or neonatal intensive care nursery admission for hypoglycaemia	816 (1 observational study)	⨁◯◯◯ Very low^b^	**OR 0.50** (0.25 to 1.00)	83 per 1,000	**40 fewer per 1,000** (61 fewer to 0 fewer)
Duration of initial hospital stay after birth	3437 (31 RCTs)	⨁◯◯◯ Very low^c,d,e^	-	Comparator	MD **2.37 days fewer** (3.66 fewer to 1.08 fewer)
Exclusive breastmilk feeding from birth to discharge	1250 (1 observational study)	⨁◯◯◯ Very low^f^	**OR 4.30** (3.19 to 5.81)	465 per 1,000	**324 more per 1,000**(270 more to 370 more)

*CI* Confidence interval, *MD* Mean difference, *OR* Odds ratio, *RR* Risk ratio

Explanations

^a^Downgraded two levels of risk of bias as four of the seven included studies were at unclear risk of selection bias, one study was at high risk of selection bias, none of the seven studies provided a detailed protocol for measuring blood glucose or explained how blood glucose was measured, and only four of the studies provided a definition for neonatal hypoglycaemia

^b^Downgraded one level for imprecision due to insufficient sample size

^c^Downgraded two levels for risk of bias as 25 out of 31 studies were at high risk of bias for at least one domain

^d^Downgraded two levels for inconsistency due to unexplained substantial heterogeneity (I^2^ = 90%)

^e^Downgraded one level for publication bias due to asymmetry in the funnel plot (p = 0.0159 Egger's test)

^f^Downgraded two levels for risk of bias as overall study quality was weak

## Discussion

### Summary of main results

This systematic review included 108 studies of 84,900 infants investigating the effect of skin-to-skin contact versus standard care or other treatment. Evidence from seven studies suggested that skin-to-skin contact may result in a large reduction in the incidence of neonatal hypoglycaemia. In addition, this review found that skin-to-skin contact reduces the incidence of hyperthermia and increases blood glucose concentrations. Skin-to-skin contact may increase the rate of exclusive breastmilk feeding within the period of 3 to 6 months and likely results in a slight increase in any breastmilk feeding at term equivalent age. The effect of skin-to-skin contact was very uncertain for admission to special care or neonatal intensive care nursery, admission to special care or neonatal intensive care nursery for hypoglycaemia, hypothermia, duration of initial hospital stay after birth, exclusive breastmilk feeding from birth to discharge, exclusive breastmilk feeding at discharge, exclusive breastmilk feeding at term equivalent age, exclusive breastmilk feeding within the period from discharge to 3 months, any breastmilk feeding at discharge, and any breastmilk feeding within the period from discharge to 3 months.

Previous research has demonstrated benefits of skin-to-skin contact for both mothers and infants. A 2021 systematic review found a beneficial effect of skin-to-skin contact on maternal anxiety and stress levels [41]. In addition, a 2023 scoping review [42] found that skin-to-skin contact improves health outcomes in the third stage of labour, including reducing post-partum haemorrhage. A 2016 [28] systematic review suggested improved cardio-respiratory stabilisation for infants who received skin-to-skin contact after birth compared to the standard care group. In addition, this review found higher blood glucose concentrations post birth in the skin-to-skin group compared to the control group but no difference in infant axillary temperatures. They found an increased rate of breastfeeding between one to four months after birth in infants who had received skin-to-skin contact compared to standard care and an increased rate of exclusive breastfeeding from discharge to one month post birth and from three months to six months post birth. Therefore, their conclusions were congruent with the findings of our review. In addition, a 2016 systematic review of KMC in low birthweight infants demonstrated a reduction in mortality, nosocomial infection, sepsis, hypothermia and an increase in weight gain, head circumference and exclusive or any breastfeeding at discharge and follow-up [29]. Alongside these previously established benefits, our review also provides evidence supporting the use of skin-to-skin contact to prevent neonatal hypoglycaemia.

This review was not specifically focused on infants at risk of neonatal hypoglycaemia. However, many of the studies were conducted in at-risk populations, mainly those born preterm. All participants in 53 of the 108 studies (49%) were at risk of neonatal hypoglycaemia due to being preterm or having a birth weight < 2.8 kg; a total of 8152 infants, or 9.5% of the infants included in the review. However, only two subgroup interactions were seen for preterm versus term infants. Preterm infants experienced increased benefit of skin-to-skin contact on exclusive breastmilk feeding from discharge to 3 months and term infants experienced increased benefit of skin-to-skin contact on any breastmilk feeding after discharge.. This suggests the continued need for other interventions that reduce the incidence of neonatal hypoglycaemia in at-risk infants.

### Possible mechanisms

There are several mechanisms through which skin-to-skin contact may reduce the incidence of neonatal hypoglycaemia. Skin-to-skin contact is suggested to assist thermoregulation of the infant by promoting vasodilation of the mother’s cutaneous blood vessels [43], thereby increasing the mother’s skin temperature. This provides heat to the infant via conduction [44] and reduces heat loss from infant to mother [45]. This means that less energy is required to maintain the infant’s body temperature. Other ways that skin-to-skin contact may reduce infant energy expenditure include reducing crying [10] and promoting quiet sleep [9], an effect that persists for at least 4 h following skin-to-skin contact. Skin-to-skin contact is also suggested to promote early breastfeeding initiation [11], which may help to prevent hypoglycaemia by enhancing provision of metabolic substrates to the infant. These mechanisms may also provide insight into the other findings of this review, including reduced risk of hyperthermia and increased rate of exclusive breastmilk feeding before discharge [11].

### Strengths and limitations of this review

#### Overall completeness and applicability of evidence

The evidence included in this review directly addresses most of the pre-specified outcomes, including the primary outcome of neonatal hypoglycaemia. However, no studies reported a number of pre-specified secondary outcomes. In addition, the included studies were from 38 countries, meaning that the findings are likely to be applicable across many cultural contexts, but no data were reported on indigenous populations including Māori. Further, only 8 studies were conducted in low-income countries, so overall findings may not be as generalisable to low-income countries.

#### Quality of the evidence

Most studies in this review were at high or unclear risk of bias in some domains. Due to the nature of the intervention, it was difficult to blind participants in RCTs and quasi-RCTs. However, many studies did not report whether outcome assessors were blinded. Lack of blinding is less of an issue for outcomes with objective measurements such as blood glucose concentration but is an issue for outcomes that can be affected by knowledge of the participants’ exposure status, such as duration of initial hospital stay after birth. Many RCTs and quasi-RCTs had unclear funding sources, meaning that it could not be determined whether the funders may have biased the methodology or reporting of outcomes. Because many of the RCTs were not pre-registered and had no published protocols, it was difficult to assess whether there was selective reporting. In addition, none of the RCTs described how neonatal hypoglycaemia was measured, so the accuracy of the methods used could not be considered. Future RCTs should report this information so that a more robust conclusion can be drawn. For the non-randomised studies of intervention, cohort studies and case–control studies, common methodological limitations were poor or no confounder adjustment, insufficient detail regarding data collection methods and loss to follow-up.

The certainty of evidence was low for neonatal hypoglycaemia and very low for special care or neonatal intensive care nursery admission for hypoglycaemia, duration of initial hospital stay after birth and exclusive breastmilk feeding from birth to discharge. All outcomes with more than 10 studies had significant publication bias demonstrated by asymmetry in the funnel plots (Additional file 5). These outcomes were hypothermia, duration of initial hospital stay, exclusive breastmilk feeding at discharge and exclusive breastmilk feeding from discharge to 3 months. This indicates that there were likely some smaller studies that were not published that may have changed the findings of the review for these outcomes.

Another reason for the low certainty of evidence was heterogeneity for some outcomes, particularly duration of initial hospital stay after birth, hypothermia, exclusive breastfeeding at discharge and any breastfeeding from discharge to 3 months. We were able to explain some of this heterogeneity with subgroup analyses. The incidence of hypothermia was only reduced if the duration of skin-to-skin contact was greater than 60 min. In addition, skin-to-skin increased blood glucose concentrations if it was initiated within 10 min of birth but not if it was initiated after 24 h. This suggests that initiating skin-to-skin contact within 10 min after birth and continuing for at least 60 min may be most effective to prevent neonatal hypoglycaemia. This is consistent with the period of the neonatal metabolic transition, during which neonatal hypoglycaemia is most common.

Preterm and term infants experienced different degrees of benefit of skin-to-skin contact on exclusive breastmilk feeding between discharge and 3 months and any breastmilk feeding after discharge. This may relate to the many other factors affecting feeding in preterm infants, including breastmilk supply [46] and infant ability to suck [47].

Skin-to-skin contact reduced the incidence of neonatal hypoglycaemia and increased blood glucose concentrations in infants delivered vaginally but not in those delivered by Caesarean section. The reasons for these differences are unclear, but potentially, the stress of vaginal birth may deplete infant glycogen stores, leaving vaginally born infants at higher risk of neonatal hypoglycaemia and therefore experiencing greater benefit from skin-to-skin contact.

#### Quality of the review

This is the first systematic review to investigate the impact of skin-to-skin contact on neonatal hypoglycaemia. It included data from 84,900 participants, comprising 108 studies, including 65 RCTs contributing evidence from 8460 infants. The large sample of infants and the large number of RCTs included are key strengths of this review. We also used a broad search strategy, including all studies that involved skin-to-skin contact, whether this was immediately after birth or as part of KMC. This ensured all relevant studies were included and we were able to obtain data for most of the identified studies. By including secondary outcomes such as hypothermia and breastmilk feeding, we were able to explore potential mechanisms for a link between skin-to-skin contact and hypoglycaemia. We were also able to examine other potential harms and adverse effects of skin-to-skin contact to aid healthcare decision-making.

A key limitation of this review is that few RCTs addressed our primary outcome of neonatal hypoglycaemia (6 RCTs, 1 quasi-RCT), and the included observational studies are at a greater risk of confounding. There is also no standard way of conducting or reporting observational studies. Because of this, the results for randomised studies and observational studies were presented separately. Another limitation is that we were unable to carry out sensitivity analysis for GRADE outcomes where significant heterogeneity was observed, because for the outcome duration of initial hospital stay after birth, only one of 32 RCTs was assessed to be high quality. In addition, for the outcomes exclusive breastmilk feeding within the period from discharge to 3 months and exclusive breastmilk feeding within the period 3 months to 6 months, some results were reported at corrected age, others at postnatal age, and in some cases the age used was not stated.

## Conclusions

Skin-to-skin contact promotes infant physiological stability (4), early (6) and exclusive (4) breastfeeding and parent-infant bonding (4).This review demonstrates that, in addition to these previously established benefits, skin-to-skin contact may lead to a large reduction in the incidence of neonatal hypoglycaemia, with low certainty of evidence. It may also reduce admission to special care and neonatal intensive care nurseries for neonatal hypoglycaemia and duration of initial hospital stay after birth and increase rates of exclusive breastmilk feeding from birth to discharge. Skin-to-skin contact is a cost-effective and accessible intervention that may be used alongside other strategies for the prevention of neonatal hypoglycaemia, as well providing other benefits for infants.

### Supplementary Information

**Additional file 1. **PRISMA checklist

**Additional file 2. **Protocol

**Additional file 3. **Search strategy

**Additional file 4: Supplementary Table 1. **Characteristics of studies table

**Additional file 5. **Funnel plots

## Data Availability

The datasets used and/or analysed during the current study are available from the corresponding author on reasonable request.

## Abbreviations

RCT: Randomised controlled trial
KMC: Kangaroo mother care
CENTRAL: Cochrane Central Register of Controlled Trials
WHO: World Health Organisation
ICTRP: International Clinical Trials Registry Platform
EPHPP: Effective Public Health Practice Project
RoB: Cochrane risk of bias tool
GRADE: Grading of Recommendations Assessment, Development and Evaluation
GDT: Grade Pro Guideline Development Tool
RR: Relative risk
OR: Odds ratio
MD: Mean difference
