# Supplementary material for: Skin-to-skin contact for the prevention of neonatal hypoglycaemia: a systematic review and meta-analysis
Source: BMC Pregnancy Childbirth. 2023 Oct 21;23:744. doi: 10.1186/s12884-023-06057-8 (PMC10590034; doi:10.1186/s12884-023-06057-8)
Supplement: Supplementary file 2 — Additional file 2. Protocol [file 12884_2023_6057_MOESM2_ESM.docx]

**Protocol - Skin-to-skin contact for the prevention of neonatal hypoglycaemia: a systematic review and meta-analysis**

**Background**

Neonatal hypoglycaemia is the most common metabolic problems among newborn babies, affecting around 5% to15% of all birth [1] and around 50% of at-risk babies [2]. Infants born of mother with diabetes, small or large for gestational age or preterm are at greatest risk [3]. Neonatal hypoglycaemia has been reported to be associated with poor neurodevelopment outcomes [4] and brain damage [5, 6]. However, some neonatal hypoglycaemia is preventable.

The UNICEF UK’s Baby Friendly Initiative recommends immediate skin-to-skin contact (SSC) between the mother and the baby as standard practice after the birth [7]. SSC involves placing the naked baby prone on the mother's bare chest for at least an hour or until after the first feed [7, 8]. Multiple studies have suggested that early SSC improves physiologic stability, including temperature and blood glucose concentrations, particularly in babies born preterm or low-birth weight [8-11]. The purpose of this systematic review is to summarise the available evidence of the effects of SSC on neonatal hypoglycaemia.

**Method**

**Searches**

We will search the following electronic bibliographic databases from inception to the April 2022: OvidMedline, Embase, CINAHL Plus and the Cochrane Central Register of Controlled Trials (CENTRAL).

We will also search for registered trials in: Current Controlled Trials (www.controlled-trials.com), Clinical Trials (www.ClinicalTrials.gov), Australian and New Zealand Clinical Trials Registry (www.anzctr.org.au), and WHO ICTRP Search Portal (https://apps.who.int/trialsearch/).

Conference abstracts will be included if they provided usable summary data.

There will be no language restrictions. The searches will be re-run just before the final analyses and further studies retrieved for inclusion. We will record the selection process in sufficient detail to complete a PRISMA flow diagram [12].

**Types of study to be included**

We will include published and unpublished randomised controlled trials (RCTs), quasi-RCTs, non-randomised studies of interventions, cohort, or case-control studies.

**Participants/population**

Pregnant women and their babies

**Intervention(s)/exposure(s)**

Standard care with skin-to-skin contact (study defined)

**Comparator(s)/control**

Standard care without skin-to-skin contact

**Outcome**

The primary outcome is neonatal hypoglycaemia (study-defined).

The secondary outcomes are:

Infants:

1. Hypoglycaemia (any blood glucose concentration < 2.6 mmol/L) during the initial hospital stay
2. Receipt of treatment for hypoglycaemia (study‐defined, any treatment including: oral dextrose gel, intravenous dextrose, or other drug therapy) during initial hospital stay
3. Number of episodes of hypoglycaemia (study‐defined)
4. Severity of hypoglycaemia (any blood glucose concentration < 2.0 mmol/L or study-defined)
5. Admission to special care nursery or neonatal intensive care nursery
6. Special care nursery or neonatal intensive care nursery admission for hypoglycaemia
7. Hypoglycaemic injury on brain imaging
8. Hyperthermia (study‐defined)
9. Hypothermia (study‐defined)
10. Duration of initial hospital stay
11. Breastmilk feeding exclusively from birth to discharge
12. Breastmilk feeding exclusively at discharge
13. Adverse effects (study-defined)

**Data extraction**

Two review authors will independently evaluate and appraise the retrieved studies using Covidence (https://www.covidence.org/), and extract data using a pre-specified data extraction form. Any disagreements will be resolved by discussion and if necessary in discussion with a third review author.

**Risk of bias (quality) assessment**

Two review authors will independently assess the risk of bias. All included RCTs or quasi-RCTs will be assessed by using the Cochrane 'Risk of bias' tool [13]: for the following domains: (1) sequence generation (selection bias); (2) allocation concealment (selection bias); (3) blinding of participants and personnel (performance bias); (4) blinding of outcome assessment (detection bias); (5) incomplete outcome data (attrition bias); (6) selective reporting (reporting bias); (7) any other bias.

Non-randomised studies of intervention will be assessed by using the ROBINS-I tool [14].

Cohort or case-control studies will be assessed by using the Newcastle-Ottawa Scale (NOS) [15].

Any disagreements will be resolved by discussion and if necessary in discussion with a third review author.

Grading of Recommendations Assessment, Development and Evaluation (GRADE) [16] approach will be used to assess the quality of evidence for the outcomes to be reported and the GradePro GDT Guideline Development Tool (https://gradepro.org/) will be used to create a ‘Summary of judgement’ table to report the quality of the key outcomes. The GRADE approach would mark the quality of a body of evidence at one of the four levels- Trivial, Small, Moderate, Large [13, 16].

The outcomes to be included for GRADE assessment are:

1. Neonatal hypoglycaemia (study-defined).
2. Receipt of treatment for hypoglycaemia (study‐defined, any treatment including: oral dextrose gel, intravenous dextrose, or other drug therapy) during initial hospital stay
3. Special care nursery or neonatal intensive care nursery admission for hypoglycaemia
4. Hypoglycaemic injury on brain imaging
5. Duration of initial hospital stay
6. Breastmilk feeding exclusively from birth to discharge

**Strategy for data synthesis**

We will consider whether the characteristics of included studies are sufficiently similar for meta-analysis. Meta-analyses will be undertaken using R, RevMan 5.4 or STATA. We will calculate I² and χ² for each analysis and describe the percentage of variability in effect estimates that may be due to heterogeneity. If we observe substantial heterogeneity (I² >50% and P < 0.10 in the χ² test), we will explore the possible causes in sensitivity analyses.

The relative risks (RRs) or odds ratios (ORs) or adjusted odds ratios (aORs) with 95% confidence intervals (CIs) will be calculated for dichotomous outcomes. The mean differences (MDs) or adjusted mean differences (aMDs) with 95% CIs will be calculated for continuous outcomes. A p-value < 0.05 will denote statistical significance.

We will assess publication bias by visual inspection of a funnel plot, plotting the study effect size against the sample size, if there are enough studies (10 or more trials). If asymmetry is apparent, we will consider and discuss possible reasons for it.

Direction of the finding tables will be used to summarise the evidence if meta-analysis is not possible.

**Subgroup analysis**

We will conduct the subgroup analyses for the following subgroups:

1. Duration of skin-to-skin contact (< 60 minutes versus ≥ 60 minutes)
2. Timing of initiation (immediate ≤10 minutes after birth versus delayed > 10 minutes)
3. Babies born preterm versus at term
4. Babies at risk of hypoglycaemia versus not at risk
5. Single versus multiple birth
6. Vaginal birth versus caesarean birth
7. Skin-to-skin contact with mother versus skin-to-skin contact with another person

**Discussion**

This systematic review and meta-analysis, using the available evidence, will assess the effect of skin-to-skin contact on prevention of neonatal hypoglycaemia. The findings may support the development of clinical guidelines for the management of neonatal hypoglycaemia.

**References**

1. Hay WW, Jr., Raju TN, Higgins RD, Kalhan SC, Devaskar SU. Knowledge gaps and research needs for understanding and treating neonatal hypoglycemia: workshop report from Eunice Kennedy Shriver National Institute of Child Health and Human Development. Journal of Pediatrics. 2009;155(5):612-7. doi:10.1016/j.jpeds.2009.06.044

2. Harris DL, Weston PJ, Harding JE. Incidence of neonatal hypoglycemia in babies identified as at risk. Journal of Pediatrics. 2012;161(5):787-91. doi:10.1016/j.jpeds.2012.05.022

3. Edwards T, Harding JE. Clinical Aspects of Neonatal Hypoglycemia: A Mini Review. Frontiers in Pediatrics. 2021;8. doi:ARTN 562251

10.3389/fped.2020.562251

4. Lucas A, Morley R, Cole TJ. Adverse neurodevelopmental outcome of moderate neonatal hypoglycaemia. BMJ. 1988;297(6659):1304-8. doi:10.1136/bmj.297.6659.1304

5. Burns CM, Rutherford MA, Boardman JP, Cowan FM. Patterns of cerebral injury and neurodevelopmental outcomes after symptomatic neonatal hypoglycemia. Pediatrics. 2008;122(1):65-74. doi:10.1542/peds.2007-2822

6. Filan PM, Inder TE, Cameron FJ, Kean MJ, Hunt RW. Neonatal hypoglycemia and occipital cerebral injury. J Pediatr. 2006;148(4):552-5. doi:10.1016/j.jpeds.2005.11.015

7. UK U. Skin-to-skin contact United Kindom [cited 2022 29/03]. Available from: <https://www.unicef.org.uk/babyfriendly/baby-friendly-resources/implementing-standards-resources/skin-to-skin-contact/>

8. Moore ER, Bergman N, Anderson GC, Medley N. Early skin-to-skin contact for mothers and their healthy newborn infants. Cochrane Database of Systematic Reviews. 2016(11). doi:10.1002/14651858.CD003519.pub4

9. Chiruvolu A, Miklis KK, Stanzo KC, Petrey B, Groves CG, McCord K, et al. Effects of Skin-to-Skin Care on Late Preterm and Term Infants At-Risk for Neonatal Hypoglycemia. Pediatr Qual Saf. 2017;2(4):e030. doi:10.1097/pq9.0000000000000030

10. Boundy EO, Dastjerdi R, Spiegelman D, Fawzi WW, Missmer SA, Lieberman E, et al. Kangaroo Mother Care and Neonatal Outcomes: A Meta-analysis. Pediatrics. 2016;137(1). doi:10.1542/peds.2015-2238

11. Dalsgaard BT, Rodrigo-Domingo M, Kronborg H, Haslund H. Breastfeeding and skin-to-skin contact as non-pharmacological prevention of neonatal hypoglycemia in infants born to women with gestational diabetes; a Danish quasi-experimental study. Sexual & Reproductive Healthcare. 2019;19:1-8. doi:10.1016/j.srhc.2018.10.003

12. Moher D, Liberati A, Tetzlaff J, Altman DG, Group P. Preferred reporting items for systematic reviews and meta-analyses: the PRISMA statement. PLoS Medicine. 2009;6(7):e1000097. doi:10.1371/journal.pmed.1000097

13. Higgins JPT, Thomas J, Chandler J, Cumpston M, Li T, Page MJ, et al. Cochrane Handbook for Systematic Reviews of Interventions version 6.3 (updated February 2022). 2022. Available from: [www.training.cochrane.org/handbook](file:///\\files.auckland.ac.nz\myhome\FMHSfiles\Guidelines\Systematic%20reviews\Skin\www.training.cochrane.org\handbook).

14. Sterne JA, Hernan MA, Reeves BC, Savovic J, Berkman ND, Viswanathan M, et al. ROBINS-I: a tool for assessing risk of bias in non-randomised studies of interventions. BMJ. 2016;355:i4919. doi:10.1136/bmj.i4919

15. Wells G, Shea B, O’Connell D, Peterson J, Welch V, Losos M, et al. The Newcastle-Ottawa Scale (NOS) for assessing the quality of nonrandomised studies in meta-analyses. Ottawa Hospital Research Institute 2013 [August 25, 2021]. Available from: <http://www.ohri.ca/programs/clinical_epidemiology/oxford.asp>

16. Schünemann H, Brozek J, Guyatt G, Oxman A, editors. GRADE handbook for grading quality of evidence and strength of recommendations. Updated October 2013. 2013. Available from: guidelinedevelopment.org/handbook
