# Supplementary material for: Skin-to-skin contact for the prevention of neonatal hypoglycaemia: a systematic review and meta-analysis
Source: BMC Pregnancy Childbirth. 2023 Oct 21;23:744. doi: 10.1186/s12884-023-06057-8 (PMC10590034; doi:10.1186/s12884-023-06057-8)
Supplement: Supplementary file 3 — Additional file 3. Search strategy [file 12884_2023_6057_MOESM3_ESM.docx]

EMBASE (1980- )

1. exp kangaroo care/

2. (skin to skin or skin on skin or kangaroo care or kangaroo mother care or baby on chest or maternal contact or chest to chest).mp. [mp=title, abstract, heading word, drug trade name, original title, device manufacturer, drug manufacturer, device trade name, keyword heading word, floating subheading word, candidate term word]

3. newborn/ or prematurity/ or newborn intensive care/ or newborn care/

4. (infant or infants or infant? or infantile or infancy or newborn* or new born or new borns or newly born or neonat* or baby* or babies or premature or prematures or prematurity or preterm or preterms or pre term or preemie or preemies or premies or low birth weight or low birthweight or VLBW or LBW or ELBW or NICU).mp. [mp=title, abstract, heading word, drug trade name, original title, device manufacturer, drug manufacturer, device trade name, keyword heading word, floating subheading word, candidate term word]

5. 1 or 2

6. 3 or 4

7. 5 and 6

Ovid MEDLINE

1. Kangaroo-Mother Care Method/

2. ("skin to skin" or "skin on skin" or "kangaroo care" or "kangaroo mother care" or "baby on chest" or "maternal contact" or “chest to chest”).mp. [mp=title, abstract, original title, name of substance word, subject heading word, floating sub-heading word, keyword heading word, organism supplementary concept word, protocol supplementary concept word, rare disease supplementary concept word, unique identifier, synonyms]

3. exp Infant, Newborn/

4. (baby* or babies or infant or infants or infant? or infantile or infancy or low birth weight or low birthweight or neonat* or newborn* or new born or new borns or newly born or premature or prematures or prematurity or preterm or preterms or pre term or preemie or preemies or premies or premie or VLBW or LBW or ELBW or NICU).ti,ab,kw,kf.

5. 1 or 2

6. 3 or 4

7. 5 and 6

Cochrane CENTRAL

#1 MeSH descriptor: [Kangaroo-Mother Care Method] explode all trees

#2 "skin to skin" or "skin on skin" or "kangaroo care" or "kangaroo mother care" or "baby on chest" or "maternal contact" or “chest to chest”

#3 #1 OR #2

#4 MeSH descriptor: [Infant] explode all trees

#5 baby* or babies or infant or infants or infant? or infantile or infancy or low birth weight or low birthweight or neonat* or newborn* or new born or new borns or newly born or premature or prematures or prematurity or preterm or preterms or pre term or preemie or preemies or premies or premie or VLBW or LBW or ELBW or NICU

#6 #4 OR #5

#7 #3 AND #6 in Trials

CINAHL PLUS

S1 kangaroo care or skin to skin or kangaroo mother care or skin-to-skin or chest-to-chest or skin contact **Expanders** - Apply equivalent subjects, **Narrow by SubjectMajor:**- infant, premature

S2 infants or baby or newborn or neonate  **Expanders** - Apply equivalent subjects

S3 S1 AND S2
