# Supplementary material for: Skin-to-skin contact for the prevention of neonatal hypoglycaemia: a systematic review and meta-analysis
Source: BMC Pregnancy Childbirth. 2023 Oct 21;23:744. doi: 10.1186/s12884-023-06057-8 (PMC10590034; doi:10.1186/s12884-023-06057-8)
Supplement: Supplementary file 4 — Additional file 4: Supplementary Table 1. Characteristics of studies table [file 12884_2023_6057_MOESM4_ESM.docx]

Supplementary Table 1. Characteristics of studies table

| **Author/Year** | **Country/ Study date** | **Participants** | **With Skin-to-skin contact** | **Standard care without skin-to-skin contact** | **Outcomes** |
| --- | --- | --- | --- | --- | --- |
| **Randomised controlled trials** | | | | | |
| Acharya 2014 (1) | Nepal/  06/2009 – 05/2010 | Inclusion criteria: Birth weight <2000 g  Exclusion criteria: Critically ill requiring ventilatory or ionotropic support or radiant warmer, chromosomal, and life-threatening congenital anomalies, mother critically ill  GA: Preterm. Intervention - 32.2±2.4, control - 32.5±1.9  BW: Intervention - 1390±230, control - 1460±170 | Timing: unspecified  Duration: ≥ 60 minutes  N=63 | Radiant warmer  N=63 | Hypothermia,  duration of initial hospital stay |
| Akcan 2009 (2) | Turkey/  02/2006 – 12/2006 | Inclusion criteria: Gestation 26-36 weeks  Exclusion criteria: Congenital abnormalities or sepsis, needing mechanical ventilation or surgical intervention  GA: Preterm. Intervention - 31.3±1.9, control - 31.9±1.2  BW: Intervention - 1580±490, control - 1760±560 | Timing: 0-28 days  Duration: ≥ 60 minutes  N=25 | Standard NICU care  N=25 | Duration of initial hospital stay |
| Ali 2009 (3) | India/  03/2006 – 09/2007 | Inclusion criteria: Born vaginally, birth weight 1200 -1800 g  Exclusion criteria: Major life-threatening congenital malformation, severe perinatal complications  GA: Intervention - 33.1±2.3, control - 33.6±2.3  BW: Intervention - 1610±210, control - 1620±180 | Timing: when stable  Duration:  ≥ 60 minutes  N=58 | Radiant warmers  N=56 | Hypothermia, duration of initial hospital stay, exclusive breastmilk feeding at 40 weeks post conceptional age, 3 months post conceptional age, and 6 months post conceptional age, |
| Anderson 2002 (4) | USA/  1997-2000 | Inclusion criteria: Healthy mother aged ≥ 18 years, healthy infant, gestation 32 -36 weeks, birth weight 1300 – 3000g and Apgar score > 6  Exclusion criteria: Mothers: eclampsia or uncontrolled seizure disorder, severe depression or mental illness refractory to treatment, serious substance abuse, transport to an intensive care unit post birth  Infants: severe congenital anomalies, admission to NICU, requiring supplemental oxygen via CPAP or mechanical ventilation  GA: Preterm. Intervention - 34.4±1.6, control - 34.6±1.4  BW: Intervention - 2260±430, control - 2210±390 | Timing: ≤10 minutes after birth  Duration: ≥ 60 minutes  N=52 | Incubators, warmer beds, bassinettes, held wrapped in blankets  N=48 | Admission to special care nursery or neonatal intensive care nursery, exclusive breastmilk feeding at discharge, 3 months, 6 months |
| Ayala 2021 (5) | Chile/  2009 - 2012 | Inclusion criteria: Healthy mother, uncomplicated elective Caesarean section, epidural anaesthesia, gestation 37-42 weeks and Apgar >7, first temperature minutes after birth ≥36.6°C  GA: Term. Intervention - 38.9±0.9, control - 38.7±1.7  BW: Not reported | Timing: > 10 minutes to 24 hours  Duration: ≥ 60 minutes  N=29 | Clothed in cot or in father’s arms  N=66 | Temperature |
| Ballesteros 2012 (6) | Philippines/  03/2011 – 07/2011 | Inclusion criteria: Birth weight ≤ 2000g.  Exclusion criteria: Respiratory distress, ventilatory support, life-threatening congenital anomalies or gross signs of chromosomal aberrations, mother critically ill and unable to breastfeed  GA: Preterm and term. Intervention - 35.2±1.7, control - 36.2±1.8  BW: Intervention - 1790±170, control - 1740±180 | Timing: >24 hours to discharge  Duration: ≥ 60 minutes  N=50 | In crib under heat lamp  N=50 | Hyperthermia, duration of initial hospital stay, blood glucose concentration |
| Beiranvand 2014 (7) | Iran/  07/2011 – 09/2011 | Inclusion criteria: Maternal age 18-40 years, elective caesarean section surgery, spinal anaesthesia, singleton, gestation 38-42 weeks and Apgar >7  Exclusion criteria: Mother: severe bleeding, uterine inertia, gestational diabetes, hypertension, heart disease. Infant: high risks and abnormalities, problems which needing hospitalisation  GA: Term. Intervention - 39±0.9, control - 38.8±1.0  BW: Intervention - 3240±290, control - 3220±340 | Timing:  > 10 minutes to 24 hours  Duration:  ≥ 60 minutes  N=48 | Dressed, embraced by mother  N=48 | Temperature |
| Bier 1996 (8) | USA/  09/1993 – 11/1995 | Inclusion criteria: Birth weight < 1500 g  Exclusion criteria: Mothers: history of illicit drug use, mental illness, HIV infection, medications contraindicative to breastfeeding. Infants: positive toxicologic screen for illicit drugs, drug withdrawal symptoms at birth  GA: Preterm. Intervention - 28±2 (range 24-33), control - 27±2 (24-33)  BW: Intervention - 990±280 (520-1470), control - 940±320 (350-1480) | Timing: Unspecified  Duration:  Unspecified  N=25 | Fully clothed infant wrapped in blanket cradled in mother’s arms  N=25 | Duration of initial hospital stay, any breastmilk feeding at discharge, 1 month, and 6 months after discharge, temperature |
| Boo 2007 (9) | Malaysia/  01/2002 – 10/2004 | Inclusion criteria: Stable, birth weight <1501 g, no ventilatory support other than nCPAP, inhaled oxygen of FiO2≤ 0.3 or a flow rate of <0.2 L/min via nasal prongs, tolerate enteral feeds >50% of the required fluid volume  Exclusion criteria: Lethal or major malformations, severe perinatal asphyxia with evidence of hypoxic-ischaemic encephalopathy, transfer to another hospital  GA: Not reported  BW: <1501 | Timing: Unspecified  Duration: ≥ 60 minutes  N=64 | N=62 | Duration of initial hospital stay, any breastmilk feeding at discharge, |
| Brotherton 2020 (10) | Gambia/  05/2018 – 03/2020 | Inclusion criteria: Singleton or twins with birth weight <2000 g  Exclusion criteria: major congenital malformations, severe jaundice, seizures, severely unstable  GA: Preterm and at term. Intervention - median 33 (IQR 31 - 34), control - 32 (31 - 34)  BW: <2000 | Timing: > 10 minutes to 24 hours  Duration: ≥ 60 minutes  N=138 | Incubator or radiant heater, no KMC until >24 hours after admission  N=141 | Hypothermia, duration of initial hospital stay, exclusive breastmilk feeding at discharge, |
| Bystrova 2003 (11) | Russia/  1995 – 1998 | Inclusion criteria: Healthy mothers without chronic diseases, normal pregnancy, singleton, vaginal birth, gestation >37 weeks  Exclusion criteria: Mothers: oxytocin infusion or analgesia during labour  GA: Term. Intervention - 39.4±1.0, control (mother’s arms) - 39.5±0.9, control (nursery) - 39.7±0.9)  BW: Intervention - 3570±450, control (mother’s arms) - 3460±470, control (nursery) - 3510±460 | Timing: > 10 minutes to 24 hours  Duration:  ≥ 60 minutes  N=44 | Clothed  and lying prone in mothers’ arm or dressed and kept in a cot in the nursery  N=132 | Temperature |
| Çaka 2003 (12) | Turkey/ June 2020 – November 2020 | Inclusion criteria: Gestation 28–36 weeks, weight 1000 - 2500 g during the research, stable vital signs, could consume 75% of the total protein and energy through an orogastric tube, fed with breast milk and eoprotein, no muscle relaxants, no analgesics, no sedatives, no inotropic drugs, no severe neurological disease, spontaneous breathing, mothers non-smoking and Turkish-speaking  Exclusion criteria: Intubated with pneumothorax, vomiting or bilious-stained gastric aspirate, gastrointestinal diseases, NEC, skull fracture, severe atelectasis or a history of surgery that might impact their comfort, condition preventing placement of a chest tube or parent’s/infant’s KMC position  GA: Preterm. Intervention - 32.1±2.8, control - 32.8±2.5  BW: Intervention - 1760 ± 500, control – 1900 ± 560 | Timing: >24 hours to discharge  Duration: ≥ 60 minutes  N = 84 | Prone position in an incubator  N = 84 | Duration of initial hospital stay, temperature |
| Carfoot 2004 (13) | UK/ Unspecified | Inclusion criteria: Healthy singleton with gestation >36 weeks  Exclusion criteria: Multiple pregnancy  GA: >36 weeks  BW: Not reported | Timing: > 10 minutes to 24 hours  Duration: unspecified  N=100 | Wrapped in a towel, given to mother or father  N=101 | Exclusive breastmilk feeding at 4 months post birth |
| Carfoot 2005 (14) | UK/  04/2002 – 09/2002 | Inclusion criteria: Healthy singleton with gestation >36 weeks  Exclusion criteria: Multiple pregnancy  GA: Term  BW: Not reported | Timing: ≤10 minutes after birth  Duration: Unspecified  N=102 | Dried and wrapped in a towel, handed to mother or father  N=102 | Hypothermia, temperature |
| Cattaneo 1998 (15) | Ethiopia, Indonesia, Mexico/  Unspecified | Inclusion criteria: Singleton, birth weight 1000-1999 g, no dependency on oxygen or IV fluids, ability to feed, no visible major malformation  Exclusion criteria: Infant abandoned by mother, multiple birth (if twins randomly assigned to different intervention)  GA: Intervention - 33.7±2.5, control - 34.0±2.2  BW: Intervention - 1620±240, control - 1640±250 | Timing: >24 hours to discharge  Duration: ≥ 60 minutes  N=149 | Warm room in Addis Ababa with open cribs and the possibility of rewarming in a bulb-heated cot, incubators in the other 2 hospitals  N=136 | Exclusive breastmilk feeding at discharge |
| Charpak 1997 (16) | Colombia/  09/1993 – 09/1994 | Inclusion criteria: Birth weight <2000 g, proper treatment for infection or concomitant condition, proper suck and swallow, weight gain or thriving in an incubator  Exclusion criteria: Referral to another institution, plans to leave Bogota soon, life-threatening, or major malformations, early detected major conditions arising from perinatal problems  GA: Babies born preterm and at term. Intervention - 33.6±2.5, control - 33.9±2.7  BW: Intervention - 1710±260, control - 1740±260 | Timing: >24 hours to discharge  Duration:  ≥ 60 minutes  N=396 | Incubator until able to regulate temperature and has appropriate weight gain  N=381 | Duration of initial hospital stay, exclusive breastmilk feeding at 40-41 weeks conceptual age, |
| Christensson 1992 (17) | Spain/ Unspecified | Inclusion criteria: Healthy vaginally born, term  Exclusion criteria: Pregnancies and deliveries with complications  GA: Term. Intervention - 39.6±1.1, control - 39.2±1.2  BW: Intervention - 3390±270, control - 3340±330 | Timing: ≤10 minutes after birth  Duration:  ≥ 60 minutes  N=25 | Cot care  N=25 | Blood glucose concentration, temperature |
| Christensson 1996 (18) | Sweden/  Unspecified | Inclusion criteria: Healthy mothers, uncomplicated pregnancy, elective caesarean section due to breech presentation or contraction of the pelvis or earlier caesarean section, spinal anaesthesia, term, Apgar score ≥8, approximate weight of 3000-4000 g, no visible malformation  GA: Term. Intervention - 38.1±0.5, control - 38.3±0.9  BW: Intervention - 3480±380, control – 3540±440 | Timing: > 10 minutes to 24 hours  Duration: ≥ 60 minutes  N=15 | Incubator or cot care  N=30 | Blood glucose concentration, temperature |
| Chwo 2002 (19) | Taiwan, China/ Unspecified | Inclusion criteria: Mothers: no pre-existing medical problems, willing to breast feed, no other problems that would prevent them from going to the nursery. Infants: gestation 34-36 weeks, Apgar score ≥ 7, no continuous respiratory assistance, admitted to normal newborn nursery or observation nursery  GA: Preterm  BW: Not reported | Timing: >24 hours to discharge  Duration: ≥ 60 minutes  N=17 | Mothers held their infants wrapped in blankets  N=17 | Duration of initial hospital stay, temperature |
| Dehghani 2015 (20) | Iran/ Unspecified | Inclusion criteria: Birth weight > 1800 g, gestation ≥32 weeks, no abnormalities and problems of nervous, cardiovascular and respiratory systems, no clinical instability and infections, no surgery, no exacerbated crying, not nil by mouth  GA: Preterm and term. Intervention - 34.5±2.4, control - 35.1±2.4  BW: Intervention - 2270±490, control - 2190±620 | Timing: unspecified  Duration: ≥ 60 minutes  N=27 | Conventional incubator care  N=27 | Duration of initial hospital stay, temperature |
| Edraki 2015 (21) | Iran/ Unspecified | Inclusion criteria: Infants: birth weight 1500-2500 g, gestation 33-36 weeks, Apgar score >7, no abnormalities or acute problems except prematurity, no sedative drugs, in incubators, ability to suck, mothers with physical and mental health, ≥1 week since birth  Exclusion criteria: Infant need for ventilation, CPAP, hypnotic and sedative drugs, use of gavage for nourishment, maternal mental or physical problems  GA: Preterm. Intervention - 33.4±1.5, control - 33.7±1.6  BW: Intervention - 1910±390, control - 1930±390 | Timing:  >24 hours to discharge  Duration: ≥ 60 minutes  N=32 | Conventional attachment style  N=32 | Duration of initial hospital stay |
| Fardig 1980 (22) | USA/ 05/1978 - 08/1978 | Inclusion criteria: Mothers: normal antenatal and intrapartum course, healthy infants born by vaginal delivery, birth weight ≥2500 g, gestation 38-42 weeks, Apgar ≥7  Exclusion criteria: Delivery by vacuum extraction or mid forceps rotation.  GA: Term  BW: Not reported | Timing: ≤10 minutes after birth  Duration: < 60 minutes  N=34 | Radiant heated crib  N=17 | Hypothermia |
| Gabriel 2010 (23) | Spain/ Unspecified | Inclusion criteria: Healthy mother, single pregnancy, documented prenatal care, admitted at 35–42 weeks gestation  Exclusion criteria: Fetal distress during labour, caesarean section, infant resuscitation, meconial amniotic fluid, no respiratory effort.  GA: Preterm and term. Intervention - 38.9±1.3, control - 39.1±1.2  BW: Intervention - 3176±390, control - 3300±410 | Timing: ≤10 minutes after birth  Duration: ≥ 60 minutes  N=175 | Wrapped in a blanket and given to parents  N=175 | Admission to special care nursery or neonatal intensive care nursery, hypothermia, exclusive breastmilk feeding at discharge and at 1 month of life |
| Gathwala 2010 (24) | India/  08/2005 – 11/2006 | Inclusion criteria: Birth weight ≤1800 g, Apgar ≥7, stable cardiopulmonary status, tolerating enteral feeding, maintaining temperature  Exclusion criteria: Infants with sickness, instability, major congenital malformations, mothers unwell  GA: Preterm and term. Intervention - 35.5±1.2, control - 35.0±1.1  BW: Intervention - 1690±110, control - 1690±120 | Timing: >24 hours to discharge  Duration: ≥ 60 minutes  N=50 | Warmer or incubator  N=50 | Exclusive breastmilk feeding at 3 months, temperature |
| Ghavane 2012 (25) | India/  04/2019 – 02/2011 | Inclusion criteria: Singleton infants, birth weight <1500 g, tolerating spoon feeds of 150 mL⁄ kg ⁄ day, not on oxygen or respiratory support, no apnoea for 72 h, not on IV fluids  Exclusion criteria: Infants with major malformation  GA: Preterm. Intervention - 30.8±2.1, control - 30.7±2.1)  BW: Intervention - 1170±190, control - 1200±190 | Timing: >24 hours to discharge  Duration: ≥ 60 minutes  N=71 | Incubator  N=69 | Neonatal hypoglycaemia, hypothermia, duration of initial hospital stay, exclusive breastmilk feeding at 40 weeks gestational age |
| Gouchon 2010 (26) | Italy/  02/2007 – 08/2007 | Inclusion criteria: Italian women, elective caesarean delivery, locoregional anaesthesia, birth weight >2,500 g, gestation 38-42 weeks, 1- and 5-min Apgar scores ≥7  GA: Term. Intervention - 38.6±0.5, control - 38.6±0.5  BW: Intervention - 3410±390, control - 33010±290 | Timing: > 10 minutes to 24 hours  Duration: ≥ 60 minutes  N=18 | Dressed and held by father or put in radiant warmer, taken to mothers’ room when she returned  N=17 | Exclusive breastmilk feeding at discharge, and 3 months, temperature |
| Hinduja 2014 (27) | India/ 11/2000 – 10/2001 | Inclusion criteria: Full term singleton infants born vaginally, birth weight ≥ 2,500 g  Exclusion criteria: Critically ill infants or mothers, infants with chromosomal and life-threatening congenital anomalies, or requiring transfer to another hospital  GA: Term  BW: Intervention - 2920±290, control - 2920±313 | Timing: ≤10 minutes after birth  Duration:  ≥ 60 minutes  N=60 | Warmer first, later clothed and bedded with mother  N=60 | Hypothermia, temperature |
| Kadam 2005 (28) | India/ 11/2000 – 10/2001 | Inclusion criteria: Birth weight <1800 g, Apgar ≥ 7, stable cardiopulmonary status in air, on breast feeds or expressed breast milk  Exclusion criteria: Sick and unstable infants with major congenital malformations  GA: Preterm. Intervention - 33.3±2.1, control - 34±1.7  BW: Intervention - 1470±230, control - 1460±220 | Timing: unspecified  Duration:  ≥ 60 minutes  N=44 | Radiant warmers, mothers able to handle infants  N=45 | Episodes of hypothermia, episodes of hyperthermia, duration of initial hospital stay |
| Kanodia 2016 (29) | Nepal/ Unspecified | Inclusion criteria: Stable infants, birth weight < 2000 g  GA: Not reported  BW: <2000 g | Timing: unspecified  Duration:  ≥ 60 minutes  N= unknown, 242 in total | Clothed and kept with mother, if required radiant warmer  N= unknown, 242 in total | Hypothermia (comment only), duration of initial hospital stay (comment only) |
| Koç 2017 (30) | Turkey  10/2014 – 01/2015 | Inclusion criteria: Maternal age 20-40 years, no psychological problems, vaginal birth without intervention except episiotomy, gestation >36 weeks  Exclusion criteria: Maternal health preventing breastfeeding or kangaroo care, infant develops health problem during kangaroo care  GA: Preterm and term  BW: Intervention - 3500±400, control - 3400±400 | Timing: ≤10 minutes after birth  Duration: < 60 minutes  N=60 | Radiant warmer  N=60 | Neonatal hypoglycaemia, hypothermia, hyperthermia, blood glucose concentration, temperature |
| Kristoffersen 2023 (31) | Norway 2014 – 2020 | Inclusion criteria: Preterm infants, gestation 28 **to** < 37 weeks, birth weight >1000 g. At one hospital, twins included and randomised to same intervention, at two hospitals, only singletons included  Exclusion criteria: Respiratory distress requiring >0.4 FiO2 to maintain oxygen saturation >90% after 20 min after birth, intubation and mechanical ventilation in the delivery room, major congenital malformations, maternal general anaesthesia  GA: Preterm. Intervention - 30.3±1.1, control - 30.3±1.2)  BW: Intervention - 1440±270, control - 1440±260 | Timing: > 10 minutes to 24 hours  Duration: ≥ 60 minutes  N = 51 | Incubator  N = 57 | Neonatal hypoglycaemia, hypothermia, hyperthermia, temperature (comment only) |
| Liao 2020 (32) | China  07/2017 – 08/2019 | Inclusion criteria: Infants born vaginally, birth weight 2500-4000 g, gestation 37-42 weeks, Apgar ≥ 8, no congenital malformations or other diseases. Mothers: ≤ 1,000 mL bleeding during delivery, no placenta or fetal membrane retained after delivery, breastfeeding  Exclusion criteria: Pregnancy complications, maternal infectious diseases, infant history of asphyxia  GA: Term. Intervention - 40.1±0.8, control - 40.2±0.8  BW: Intervention - 3310±370, control - 3270±340 | Timing: > 10 minutes to 24 hours  Duration: ≥ 60 minutes  N=39 | Infants clothed  N=39 | Exclusive breastmilk feeding at 42 days after birth |
| Linner 2022 (33) | Norway and Sweden  2018 -2021 | Inclusion criteria: Gestation 28-33 weeks  Exclusion criteria: Triplets or higher order births, congenital malformations that required immediate intervention, severe congenital infection  GA: Preterm. Intervention - 31+2 ± 1, range 28+6 – 32+5, control - 31+0±1, 28+4 – 32+6  BW: Intervention – 1570±400, 700–2350, control - 1490±400, 560–2440 | Timing: ≤10 minutes after birth  Duration:  ≥ 60 minutes  N=53 | Incubator or cot  N=48 | Neonatal hypoglycaemia (comment only) hypothermia, hyperthermia, adverse events (comment only) |
| Ludington-Hoe 2000 (34) | USA/ Unspecified | Inclusion criteria: Singleton, birth weight 770 - 2710 g, gestation 26-35 weeks, in incubators, no longer requiring supplemental oxygen, no significant apnoea and bradycardia  GA: Preterm. Intervention - 30.6±2.5, range 26-35, control - 29.6±1.9, 27-33  BW: Intervention - 1410±470, 910-2710, control - 1380±370, 780-2090 | Timing: >24 hours to discharge  Duration: ≥ 60 minutes  N=16 | Incubator  N=13 | Temperature |
| Ludington-Hoe 2004 (35) | USA/ Unspecified | Inclusion criteria: Stable, birth weight appropriate for gestation, gestation 32-36 weeks, in open cribs. No known conditions affect the dependent measures, no medication for at >24 hours prior to study  GA: Preterm. Intervention - 33.6±1.6, control - 34.4±1.1  BW: Intervention - 1880±520, control - 2010±350 | Timing: >24 hours to discharge  Duration: ≥ 60 minutes  N=11 | Crib covered with thermal blanket  N=13 | Temperature |
| Luong 2016 (36) | Vietnam/  12/2010 – 12/2011 | Inclusion criteria: Singleton, birth weight 1500-2490 g  Exclusion criteria: Mothers: HIV, hepatitis B, poor health. Infants: severe malformation, chromosomal abnormality, life-threatening disorders requiring complex technology, multiple births, severe asphyxia at birth, neonatal convulsions  GA: Preterm and term. Intervention - 33.6±1.8, control - 33.9±1.8  BW: Intervention - 2060±290, control – 2080±260 | Timing: ≤10 minutes after birth  Duration: Unspecified N=50 | Clothed and placed in incubator or crib  N=50 | Neonatal hypoglycaemia, hypothermia, duration of initial hospital stay, blood glucose concentration |
| Mahmood 2011 (37) | Pakistan/ 11/2009 – 12/2009 | Inclusion criteria: Healthy, full-term, born vaginally  Exclusion criteria: Mothers: multiple pregnancy, pre-existing medical complications, severe postpartum haemorrhage, caesarean section, severely retracted/inverted nipples, passage of meconium during labour. Infants: birth weight < 2500 g, respiratory distress after birth, major congenital anomalies, floppiness, birth trauma  GA: Term. Intervention - 38.9±1.3, control - 38.9±1.2  BW: Intervention - 3060±340, control - 3040±350 | Timing: ≤10 minutes after birth  Duration: unspecified  N=92 | Radiant warmer, wrapped with sheets and given to mother when mother ready  N=91 | Exclusive breastmilk feeding at 1 month follow-up |
| Mizuno 2004 (38) | Japan/ 02/2002 – 03/2002 | Inclusion criteria: Born vaginally, gestation >37 weeks, birth weight appropriate for gestational age, Apgar ≥8  Exclusion criteria: Major congenital abnormalities  GA: Term. Intervention - 39.5±1.4, control - 39.5±0.9  BW: Intervention - 3010±390, control - 3210±410 | Timing: ≤10 minutes after birth  Duration: unspecified  N=30 | Separated from their mothers in the neonatal ward for 24 h after birth  N=30 | Exclusive breastmilk feeding at discharge |
| Mohammadzadeh 2010 (39) | Iran/ Unspecified | Inclusion criteria: Preterm, birth weight < 2000 g, stabilised in first 72 h  Exclusion criteria: Ventilatory support or IV nutrition, apnoea or unstable temperature  GA: Preterm. Intervention - 30.6±3.1, control - 32.3±3.6  BW: Intervention - 1260±270.1, control - 1500±350 | Timing: >24 hours to discharge  Duration:  ≥ 60 minutes  N=50 | Incubator, mother may pick up baby  N=50 | Duration of initial hospital stay |
| Mwendwa 2012 (40) | Kenya/ 06/2005 – 03/2006 | Inclusion criteria: Stable, birth weight 1000-1750g  GA: Preterm. Intervention - 32.7 (range 28-36), control - 33.3 (26-36)  BW: Intervention - 1510 (1000-1900), control - 1540 (950-1900) | Timing: >24 hours to discharge  Duration: ≥ 60 minutes  N=85 | N=81 | Duration of initial hospital stay |
| Myron 2017 (41) | USA/ Unspecified | Inclusion criteria: Term infants  GA: Term  BW: Not reported | Timing: > 10 minutes to 24 hours  Duration: ≥ 60 minutes  N=unknown, total =120 | Radiant warmer  N=unknown, total =120 | Temperature (comment only) |
| Nimbalkar 2014 (42) | India/ 06/2012 – 03/2013 | Inclusion criteria: Stable term infant, born vaginally, birth weight ≥1800 g  Exclusion criteria: Congenital malformation or resuscitation  GA: Preterm and term (Intervention - 37.8±1.4, control - 37.7±1.3)  BW: Intervention - 2620±400, control - 2590±440 | Timing: > 10 minutes to 24 hours  Duration: ≥ 60 minutes  N=50 | Clothed, bedding in with mother  N=50 | Hypothermia, temperature |
| Ocampo 2013 (43) | Philippines/ Unspecified | Inclusion criteria: Stable, birth weight ≤1500 g, no dependency on oxygen or IV fluid, ability to feed, stable vital signs for past 24 h, no apnoea, no IV lines or well-secured peripheral line, no sepsis, on IV antibiotic therapy but clinically stable, can require phototherapy but steady total serum bilirubin level  Exclusion criteria: Chromosomal and life-threatening congenital anomalies, severe or critical illness  GA: Preterm and term. Intervention - 32.5±2.8, control - 32.1±2.5  BW: Intervention - 1170±0.19, control - 1210±0.23 | Timing: >24 hours to discharge  Duration: ≥ 60 minutes  N=26 | Clothed, placed in bassinet  N=26 | Hypothermia, duration of initial hospital stay |
| Olmedo 2012 (44) | Brazil/ 09/2009 – 10/2009 | Inclusion criteria: Gestation 24-36 weeks, breathing spontaneously, non-invasive monitoring, haemodynamically stable, hospitalisation ≤28 days  Exclusion criteria: Congenital malformations, perinatal hypoxia, developmental abnormality, invasive or non-invasive mechanical ventilation at time of collection, clinical worsening prevented data collection, discharged before end of collection  GA: Preterm  BW: Not reported | Timing: Unspecified  Duration: ≥ 60 minutes  N=10 | Heated incubator  N=10 | Temperature |
| Perez-Jimenez 2022 (45) | Spain/ 01/2019 – 11/2019 | Inclusion criteria: Maternal age 20-40 years, Caesarean section, term healthy infant, Apgar ≥9, no fetal alterations or malformations  Exclusion criteria: Caesarean section complicated by serious pathology, urgent caesarean section  GA: Term  BW: Intervention – 3620±470, control - 3510±810 | Timing: ≤10 minutes after birth  Duration: ≥ 60 minutes  N=40 | In crib  N=40 | Any breastmilk feeding at 1 month |
| Pouraboli 2019 (46) | Iran/ 2015 | Inclusion criteria: Caesarean section, spinal anaesthesia, Iranian nationality, singleton term pregnancy  Exclusion criteria: Respiratory distress, cyanosis, meconium in the amniotic fluid, neonatal anomalies, resuscitation, admission to NICU  GA: Term  BW: Not reported | Timing: > 10 minutes to 24 hours  Duration: <60 minutes  N=40 | Neonate given to father or mother’s companion  N=40 | Exclusive breastmilk feeding at discharge |
| Ramanathan 2001 (47) | India/ Unspecified | Inclusion criteria: Stable, birth weight <1500 g, enteral feeds, maintaining temperature  Exclusion criteria: Mothers unable to come to nursery  GA: Preterm. Intervention - 30.4 (range 28.8-34.1), control - 30.9 (29-33.3)  BW: Intervention - 1220±190, control - 1270±170 | Timing: Unspecified  Duration: ≥ 60 minutes  N=14 | Warmer or incubator  N=14 | Duration of initial hospital stay, exclusive breastmilk feeding at 6 weeks |
| Ramani 2015 (48) | Zambia/ Unspecified | Inclusion criteria: Term neonates  GA: Term  BW: Not reported | Timing: ≤10 minutes after birth  Duration: < 60 minutes  N=101 | N=102 | Hypothermia |
| Ricero-Luistro 2020 (49) | Philippines/ Unspecified | Inclusion criteria: Admitted to NICU, gestation 28 to 36 weeks, birth weight 1000-2000 g, Apgar > 7, nCPAP due to respiratory distress  Exclusion criteria: Major congenital abnormalities  GA: Preterm. Intervention - 32.7±2.6, control - 32.3±2.2  BW: Intervention - 1560±330.2, control - 1551.3±287.8 | Timing: >24 hours to discharge  Duration: ≥ 60 minutes  N=35 | Incubator  N=35 | Hypothermia, duration of initial hospital stay, blood glucose concentration |
| Roberts 2000 (50) | Australia/ Unspecified | Inclusion criteria: Stable, gestation ≥ 30 weeks, Apgar > 5, stable temperature for 24 h, extubated ≥48 h, ceased crib or headbox oxygen ≥24 h, cared for in open crib or incubator  Exclusion criteria: Congenital abnormalities or central nervous system impairment, phototherapy within 24 h, resuscitation, episodes of apnoea or bradycardia requiring more than mild stimulation, maternal drug use history  GA: Preterm. Intervention - 31.7±3.1, control - 31.2±2.4  BW: Intervention - 1560±470, control - 1480±410 | Timing: >24 hours to discharge  Duration:  ≥ 60 minutes  N=16 | Swaddled in infant clothing and a light blanket  N=14 | Duration of initial hospital stay, any breastfeeding at discharge, temperature (comment only) |
| Rojas 2003 (51) | USA/ 08/1995 – 04/1998 | Inclusion criteria: Birth weight ≤1500 g, gestation ≤32 weeks, minimal ventilatory support or extubated on nCPAP or nasal canula, hemodynamic stability  Exclusion criteria: Maternal age < 18 years, illicit drug use during pregnancy, perinatal asphyxia, potential transfer within first month after birth, major congenital anomaly, planned adoption, Grade III or IV intraventricular haemorrhage, birthweight <10th percentile for age, suspected sepsis  GA: Preterm. Intervention - 26.6±2.3, control - 27.2±2.3  BW: Intervention - 910±250, control - 940±230 | Timing: >24 hours to discharge  Duration:  ≥ 60 minutes  N=33 | Held in parents’ arms wrapped in a blanket  N=27 | Hypothermia, hyperthermia, adverse events (comments only) |
| Sharma 2016 (52) | India/ 05/2008 – 09/2009 | Inclusion criteria: Singleton term infants born vaginally, no resuscitation  Exclusion criteria: Major congenital malformations, breech, ventouse or forceps delivery, contraindications for breastfeeding, mothers: HIV, refuse to breastfeed  GA: Term. Intervention – mean 38 (IQR 37-39), control – 38 (37.5-39)  BW: Intervention - 2760±320 control - 2800±340 | Timing: ≤10 minutes after birth  Duration: < 60 minutes  N=100 | Radiant warmer  N=100 | Exclusive breastmilk feeding at 6 weeks, temperature |
| Sloan 1994 (53) | Ecuador/  11/1991 – 12/1992 | Inclusion criteria: Singleton, birth weight < 2000 g, temperature 36.5-37.0 C for 24 h before enrolment; tolerance of food; no decrease in weight for those weighing < 1750 g at birth for >72 h  Exclusion criteria: Infants with serious congenital abnormalities, respiratory, metabolic, or infectious disease  GA: Preterm and term. Intervention - 34.6±2.5, control - 34.1±2.4  BW: Intervention - 1708±240, control - 1700±250 | Timing:  >24 hours to discharge  Duration:  ≥ 60 minutes  N=140 | N=160 | Duration of initial hospital stay (comment only) |
| Srivastava 2014 (54) | India/ 07/2009 – 07/2011 | Inclusion criteria: Term singleton, born vaginally, no resuscitation beyond initial steps post-partum  Exclusion criteria: Major congenital malformation, separated from mother postpartum  GA: Term  BW: Intervention - 3000±350, control – 2990±400 | Timing:  Early >10 minutes to 24 hours  Duration:  ≥ 60 minutes  N=150 | Standard care (wrapped in a blanket and placed next to mother)  N=148 | Exclusive breastmilk feeding at 6 weeks, temperature |
| Suciu 2016 (55) | Romania01/2015 – 12/2015 | Inclusion criteria: Low birthweight  GA: Not reported  BW: Not reported | Timing:  Unspecified  Duration:  Unspecified  N=unknown  Total =64 | Usual hospital care  N=unknown  Total =64 | Exclusive breastmilk feeding (no time point provided so not meta-analysed), blood glucose concentration (comment only) |
| Suman 2008 (56) | India/ Unspecified | Inclusion criteria: Singleton intramural infant, birth weight <2000 g  Exclusion criteria: Critically ill requiring ventilatory or inotropic support, chromosomal and life-threatening congenital anomalies, requiring transfer, mother critically ill, unable to comply with the follow-up schedule  GA: Preterm and term. Intervention - 35.3±2.3, control - 35.9±2.1  BW: Intervention - 1680±240, control - 1720±240 | Timing: >24 hours to discharge  Duration: ≥ 60 minutes  N=108 | Servo controlled radiant warmer or cradle under hot lamp  N=112 | Neonatal hypoglycaemia, hypothermia, hyperthermia, duration of initial hospital stay |
| Thomson 1979 (57) | Canada/ Unspecified | Inclusion criteria: Born vaginally, birth weight >2500g, gestation >38 weeks, Apgar ≥8, without visible malformation  GA: Term  BW: Intervention - 3370±330, control - 3330±520 | Timing: > 10 minutes to 24 hr  Duration: < 60 minutes  N=17 | Usual hospital routine (wrapped in a blanket and held for <5 minutes by mother)  N=17 | Any breastmilk feeding at discharge |
| Thukral 2010 (58) | India/ 08/2008 – 09/2009 | Inclusion criteria: Term, appropriate-for-gestational age, born vaginally  Exclusion criteria: Major congenital anomalies, diabetic mother, required resuscitation beyond the initial steps, admission to intensive care unit  GA: Term. Intervention - 38 (range 38-39), control - 38 (37-39)  BW: Intervention - 2840±250, control - 2760±300 | Timing: ≤10 minutes after birth  Duration: ≥ 60 minutes  N=20 | Kept by the mother’s side  N=21 | Exclusive breastmilk feeding at 6 weeks |
| Villalon 1993 (59) | Chile/ 12/1991 – 03/1992 | Inclusion criteria: Born vaginally, birth weight 2500-4250 g, gestation 38-41 weeks, Apgar ≥ 4  Exclusion criteria: Membrane rupture > 12 hours, suspicion of infection, malformations, respiratory distress, heart disease, or hypoglycaemia  GA: Term. Intervention - 39.1±0.8, control - 38.3±1.0  BW: Intervention - 3440±300, control - 3350±360 | Timing: > 10 minutes to 24 hours  Duration:  Unspecified  N=59 | Care as usual  N=60 | Exclusive breastmilk feeding at discharge, and at 2 weeks, temperature |
| Wang 2021 (60) | China/ Unspecified | Inclusion criteria: Gestation 33-36 weeks, no known congenital anomalies, respiratory difficulties, mother age ≥ 18 years  Exclusion criteria: Severe or critical illness, congenital anomalies, severe periventricular/intraventricular haemorrhage, surgery  GA: Preterm  BW: Intervention - 2100±210, control - 2200±240 | Timing: Unspecified  Duration: ≥ 60 minutes  N=36 | Standard care (no contact with parents in the NICU)  N=43 | Exclusive breastmilk feeding at 6 months corrected age |
| Whitelaw 1998 (61) | UK/ 08/1985 – 02/1987 | Inclusion criteria: Birth weight <1500 g, stable breathing with no oxygen requirement, at least one parent spoke fluent English  GA: Preterm. Intervention - 29.1±2.3, control - 29.5±2.3  BW: Intervention - 1150±220, control - 1140±260 | Timing: >24 hours to discharge  Duration:  ≥ 60 minutes  N=35 | Incubator, baby clothed for cuddling  N=36 | Duration of initial hospital stay |
| Worku 2005 (62) | Ethiopia/ 11/2001 – 11/2002 | Inclusion criteria: Singleton, birth weight <2000 g, no major congenital malformation, healthy mother  GA: Preterm and term. Intervention - 32.5, control - 31.6  BW: Intervention - 1510 (range 1000–1900), control - 1470 (930–1900) | Timing: > 10 minutes to 24 hours  Duration:  Unspecified  N=62 | Artificial warming  system  N=61 | Duration of initial hospital stay |
| Xu 2019 (63) | China/  01/2018 – 12/2018 | Inclusion criteria: Preterm, caesarean section, birth weight ≥ 2000 g, gestation 34-36 weeks, Apgar ≥8, no congenital disease, no need to be transferred to NICU immediately  Exclusion criteria: Massive bleeding during caesarean section, critically ill such as eclampsia and amniotic fluid embolism; communication disorders, mental disease, combined medical and surgical diseases requiring general anaesthesia  GA: Preterm. Intervention - 35.8±0.9, control - 35.7±0.9  BW: Intervention - 2500±310, control - 2540±340 | Timing: ≤10 minutes after birth  Duration: ≥ 60 minutes  N=60 | Radiation bed, covered with a quilt  N=60 | Admission to special care nursery or neonatal intensive care nursery, hypothermia, temperature |
| Zafran 2022 (64) | Israel/ 08/2016 – 02/2018 | Inclusion criteria: Term singleton, caesarean delivery, spinal anaesthesia, maternal age 18-45 years  Exclusion criteria: Infant: unplanned caesarean delivery, major fetal malformations, requiring immediate neonatologist evaluation, estimated weight <5th percentile. Mother: uncontrolled diabetes mellitus, severe preeclampsia, maternal HIV, failed spinal anaesthesia requiring general anaesthesia  GA: Term. Intervention - 38.5±0.6, control - 38.4±0.7  BW: Intervention - 3270±480 control – 3290±480 | Timing: ≤10 minutes after birth  Duration: < 60 minutes  N=108 | Infants given to the mother for a few minutes then transferred to the nursery unit  N=106 | Neonatal hypoglycaemia, admission to special care nursery or neonatal intensive care nursery, hypothermia, exclusive breastmilk feeding at discharge |
| Zheng 2022 (65) | China/ 02/2019 – 01/2020 | Inclusion criteria: Caesarean section, Apgar ≥8  Exclusion criteria: Mother unwilling to breastfeed, gestational diabetes and hypertension, infectious diseases, resuscitation leading to separation of mother and child  GA: Not reported  BW: Intervention - 3340±370, control - 3390±335 | Timing: unspecified  Duration: ≥ 60 minutes  N=140 | Routine care  N=140 | Any breastmilk feeding after discharge |
| **Quasi-randomised controlled trials** | | | | | |
| Bose 2021 (66) | India/  unspecified | Inclusion criteria: Caesarean section  GA: Not reported  BW: Not reported | Timing: > 10 minutes to 24 hours  Duration: < 60 minutes  N=40 | No intervention  N=40 | Hypothermia, hyperthermia, temperature (comment only) |
| Britton 1980 (67) | USA/  unspecified | Inclusion criteria: Low-risk pregnancy, no labour and delivery complications, vaginal birth, gestation 37-42 weeks, birth weight >6 pounds (2700 g), Apgar ≥7; no signs of distress  GA: Term  BW: > 2700 | Timing: > 10 minutes to 24 hours  Duration:  ≥ 60 minutes  N=15 | Heated crib  N=19 | Temperature |
| Kambarami 1998 (68) | Zimbabwe/ 09/1995- 02/1996 | Inclusion criteria: Singleton, preterm, aged <7 days, birth weight <1600 g, able to suck  Exclusion criteria: Twins, major congenital malformations, recovering from major surgery  GA: Preterm  BW: Intervention - 1460 (Q1, Q3: 1400, 1450), control -  1400 (1260, 1450) | Timing: >24 hours to discharge  Duration: Unspecified  N=37 | Incubator, moved to cot if insufficient incubators  N=37 | Duration of initial hospital stay |
| Karimi 2020 (69) | Iran/ 2016 | Inclusion criteria: Birth weight < 2,500g, gestation 32-36 weeks, no congenital abnormalities, no muscular dysfunction, no phototherapy  Exclusion criteria: nCPAP  GA: Preterm  BW: <2500 | Timing: Unspecified  Duration: ≥ 60 minutes  N=50 | Conventional care in incubator  N=50 | Duration of initial hospital stay |
| Kashaninia 2015 (70) | Iran/ 2012 -2013 | Inclusion criteria: Singleton, preterm, birth weight <2500 g, Apgar ≥ 7, no congenital defect, no mechanical ventilation.  GA: Preterm  BW: Intervention - 1220±270, control - 1100±220 | Timing: >24 hours to discharge  Duration:  ≥ 60 minutes  N=23 | N=23 | Duration of initial hospital stay |
| Keshavarz 2010 (71) | Iran/ 2010 | Inclusion criteria: Maternal age 18-40 years, caesarean section, singleton  Exclusion criteria: Mother: diabetes, hypertension, cardiovascular disease, pre-eclampsia, gestational hypertension, placental abruption, mental health problem, divorce, unplanned pregnancy, meconium, drug abuse, mother doesn't like sex of baby, water breaking 17 hours before caesarean. Infant: any abnormality, Apgar <7, NICU admission  GA: Intervention - 277±5 days, control - 275±6 days  BW: Intervention - 3310±300, control - 3230±290 | Timing: > 10 minutes to 24 hours  Duration:  ≥ 60 minutes  N=80 | Routine care  N=80 | Temperature |
| Kucukoglu 2021 (72) | Turkey/ 10/2015 – 02/2017 | Inclusion criteria: Mother: no health problems including alcohol or substance addiction. Infant: gestation 28-37 weeks, Apgar ≥7, in hospital > 72 hours  Exclusion criteria: Positive pressure ventilation, congenital anomalies, infections, reduced body temperature, oxygen saturation  GA: Preterm. Intervention - 32.4±2.5, control - 32.9±2.4  BW: Intervention -2000±580, control - 2000±500 | Timing: unspecified  Duration:  ≥ 60 minutes  N=30 | Routine care  N=30 | Duration of initial hospital stay, exclusive breastmilk feeding at discharge, 3 months and 6 months after discharge |
| Mathews 2018 (73) | India/ Unspecified | No description of inclusion/exclusion criteria  GA: Not reported  BW: Not reported | Timing: unspecified  Duration: ≥ 60 minutes  N=20 | N=20 | Temperature |
| Mehrpisheh 2022 (74) | Iran/ 03/2019 – 02/2020 | Inclusion criteria: Singleton, gestation < 37 weeks, admitted to NICU, no birth defects, no mechanical ventilation, no surgery, no history of maternal mental disorder  GA: Preterm. Intervention - 32.1±2.9, control - 33.2±2.8  BW: Intervention - 1460±360, control - 1490±320 | Timing: >24 hours to discharge  Duration: ≥ 60 minutes  N=50 | Normal care  N=50 | Duration of initial hospital stay |
| Parsa 2018 (75) | Iran/ 02/2016 – 09/2016 | Inclusion criteria: Birthweight < 2500 g, gestation 34-36 weeks  Exclusion criteria: Infant: too sick, mechanical ventilation. Mother: illness or complications that prevent caring for baby  GA: Preterm  BW: <2500 | Timing: unspecified  Duration:  ≥ 60 minutes  N=50 | Incubator  N=50 | Temperature |
| Rangey 2014 (76) | India/ 2013 | Inclusion criteria: Medically stable, birthweight <2500g, gestation <37 weeks  Exclusion criteria: Congenital, orthopaedic, genetic abnormalities, ventilated  GA: Preterm  BW: <2500 | Timing: Unspecified  Duration: ≥ 60 minutes  N=15 | Field massage therapy involving tactile stimulation and kinaesthetic stimulation  N=15 | Duration of initial hospital stay, temperature |
| Rehman 2020 (77) | Pakistan/ 03/2018 – 10/2018 | Inclusion criteria: Infants in NICU  Exclusion criteria: Necrotising enterocolitis, mechanical ventilation or surgical intervention, left against medical advice before completion of data-collection process  GA: Preterm and at term. Intervention - 35.1±2.7, control - 36.2±2.4  BW: Not reported | Timing: > 24 hours to discharge  Duration: ≥ 60 minutes  N=70 | N=70 | Duration of initial hospital stay |
| Safari 2018 (78) | Iraq  02/2017 – 05/2017 | Inclusion criteria: Born vaginally, gestation 38-42 weeks, Apgar >7, normal pregnancy  GA: Term  BW: Not reported | Timing: ≤10 minutes after birth  Duration: ≥ 60 minutes  N=56 | N=52 | Hypothermia, temperature |
| Solanki 2021 (79) | India/ Unspecified | No description  GA: Preterm  BW: Not reported | Timing: unspecified  Duration: Unspecified  N=30 | N=30 | Temperature (comment only) |
| Swarnkar 2016 (80) | India/ 05/2015 – 10/2015 | Inclusion criteria: Singleton intramural infant, birth weight <2500 g  Exclusion criteria: Infant: Critically ill requiring ventilatory or inotropic support, chromosomal and life-threatening congenital anomalies, requiring transfer. Mother: critically ill  GA: Preterm and term. Intervention - 35.5±2.4, control - 35.9±2.4  BW: <2500 | Timing: > 24 hours to discharge  Duration: ≥ 60 minutes  N=30 | Servo controlled radiant warmers, cradle under hot lamps in NICU  N=30 | Neonatal hypoglycaemia, hypothermia, hyperthermia, duration of initial hospital stay |
| Toprak 2022 (81) | Turkey/ 07/2015 – 01/2016 | Inclusion criteria: Birth weight 2500-4000g, Apgar ≥7, no congenital abnormalities, 95% oxygen saturation after birth  GA: Intervention - 273±6 days, control - 272±5 days  BW: Intervention - 3380±380, control - 3470±350 | Timing: ≤10 minutes after birth  Duration: < 60 minutes  N=34 | N=39 | Temperature |
| **Non-randomised studies of intervention** | | | | | |
| Albuquerque 2016 (82) | Brazil  02/2013 – 03/2014 | Inclusion criteria: Maternal age ≥ 18 years, gestation 37-41 weeks, Apgar scores >7  Exclusion criteria: Complications at delivery, foetal distress, neonatal reanimation  GA: Babies born at term  BW: Not reported | Timing: ≤10 minutes after birth  Duration: Not specified  N=32 | Radiant heat  N=29 | Hypothermia, temperature |
| Ferrara 2017 (83) | Uganda  03/2014 – 06/2014 | Inclusion criteria: Healthy term infant, Apgar >5  GA: Term. Intervention - 39.5±1.7 (range 36-43), control - 39 ±2.4 (34-44)  BW: Intervention - 3.11±0.40 kg, control - 3.06±0.39 kg | Timing: ≤10 minutes after birth  Duration: ≥ 60 minutes  N=66 | Heater  N=65 | Neonatal hypoglycaemia,  hypothermia |
| Gonuguntla 2018 (84) | India  Unspecified | Inclusion criteria: Birth weight <2000g  GA: Preterm and term  BW: <2000 | Timing: when the infants are stable  Duration: ≥ 60 minutes  N=90 | Wrapped appropriately and placed next to the mother  N=60 | Hypothermia |
| Kristoffersen 2016 (85) | Norway  04/2010 – 05/2013 | Inclusion criteria: Stable, preterm, born vaginally, gestation 32-34 weeks  Exclusion criteria: Major congenital malformations.  GA: Babies born preterm. Intervention – median (range) 33+5 (32+0–34+6), control (Vestfold Hospital) – 34+2 (32+0-34+6), control (University Hospital of North Norway) - 34+2 (32+0-34+6)  BW: Intervention – median (range) 2,100 (1,500–2,830), control (Vestfold Hospital) – 1,980 (1,590–3,280), control (University Hospital of North Norway) - 2,080 (1,570–2,760) | Timing: ≤10 minutes after birth  Duration: ≥ 60 minutes  N=50 | Incubator  N=49  (Vestfold Hospital n = 25, University Hospital of North Norway n = 18) | Hypothermia |
| Li 2022 (86) | China  12/2019 – 12/2020 | Inclusion criteria: Stable, preterm, gestation 34-36 weeks or stable, term, low birthweight  GA: Preterm  BW: Not reported | Timing: ≤10 minutes after birth  Duration: ≥ 60 minutes  N=186 | N=161 | Temperature |
| Shattnawi 2019 (87) | Jordan  03/2017 - 08/2017 | Inclusion criteria: Mother: no critical illnesses. Infant: stable, birth weight >1800g, gestation 26-37 weeks  GA: Preterm. Intervention - 32±2.48, control - 33±2.41  BW: Not reported | Timing: unspecified  Duration: ≥ 60 minutes  N=48 | Incubator care  N=41 | Duration of initial hospital stay, exclusive breastmilk feeding at discharge, |
| Yin 2000 (88) | China  02/1998 – 05/ 1998 | Inclusion criteria: Birth weight < 2000g, no respiration support, no congenital disease, stable  GA: Preterm  BW: <2000 | Timing: unspecified  Duration: < 60 minutes  N=18 | N=18 | Temperature |
| **Prospective cohort studies** | | | | | |
| Casolino 2022 (89) | Italy  Unspecified | Inclusion criteria: Healthy, term, vaginal delivery  GA: Term  BW: Not reported | Timing: ≤10 minutes after birth  Duration: ≥ 60 minutes  N=79 | Routine care  N=81 | Exclusive breastmilk feeding at 3 months |
| Guala 2017 (90) | Italy  Unspecified | Inclusion criteria: Term, caesarean section, Apgar >7  GA: Term  BW: Not reported | Timing: ≤10 minutes after birth  Duration: ≥ 60 minutes  N=189 | Care with heater  N=63 | Exclusive breastfeeding at discharge, at 3 months, and 6 months |
| Juan 2022 (91) | China  01/2021 – 08/2021 | Inclusion criteria: Singleton, term, elective caesarean section  GA: Babies born at term. Intervention - median (IQR) - <30 min: 39.3 (38.9–39.6), 30-59 min: 39.10 (38.70–39.40), 60-89 min: 39.0 (38.7–39.5), ≥ 90 min: 39.0 (38.7–39.4), control - 39.14 (38.7-39.4)  BW: Intervention - <30 min: 3520±440, 30-59 min: 3410±380, 60-89 min: 3440±440, ≥90min: 3400±390 control - 3360±400 | Timing: ≤10 minutes after birth  Duration: <30 min n = 84, 30-59 min n = 168, 60-89 min n = 146, ≥90 min n = 145  N=543 | Standard care  N=136 | Admission to special care nursery or neonatal intensive care nursery, duration of initial hospital stay, exclusive breastmilk feeding at discharge |
| Lamy Filho 2008 (92) | Brazil  03/2004- 03/2005 | Inclusion criteria: Birth weight 500-1749 g.  Exclusion criteria: Major malformations, congenital TORCH infection (toxoplasmosis, rubella, cytomegalovirus, herpes simplex or syphilis), chromosomopathies, metabolic abnormalities, severe perinatal encephalopathy, necrotising enterocolitis requiring surgery  GA: Preterm. Intervention - 32.4±2.3, control - 31.7±2.8  BW: Intervention -1400±260, control - 1310±300 | Timing: Unspecified  Duration: Unspecified  N=621 | N=366 | Duration of initial hospital stay, exclusive breastmilk feeding at discharge, |
| Mikiel-Kostyra 2002 (93) | Poland/  Unspecified | Inclusion criteria: Birth weight ≥2500 g,gestation ≥ 37 weeks, without birth asphyxia, disorders of adaptation and visible congenital malformations, discharge at 2–15 d after birth  GA: Term  BW: ≥2500 | Timing: ≤10 minutes after birth  Duration: < 60 minutes  N=1020 | N=230 | Exclusive breastmilk feeding at from birth to discharge |
| Ruiz 2021 (94) | Spain/  Unspecified | Inclusion criteria: Gestation 34-42 weeks, no IV glucose solution to mother during delivery  GA: Preterm and term  BW: Not reported | Timing: Unspecified  Duration: Unspecified  N=149 | Without SSC  N=48 | Neonatal hypoglycaemia |
| Suzuki 2013 (95) | Japan  02/2011 – 11/2011 | Inclusion criteria: Singleton, born vaginally, gestation 37 to 41 weeks  GA: Term. Intervention - 39.4± 1.3, control - 39.1± 1.5 BW: Intervention - 3030± 280, control - 3030± 470 | Timing: ≤10 minutes after birth  Duration: ≥ 60 minutes  N=272 | Without early SSC  N=131 | Exclusive breastmilk feeding 1 month after delivery |
| Zhang 2020 (96) | China  03/2018 – 03/2019 | Inclusion criteria: Gestation 34-37 weeks  GA: Preterm  BW: Not reported | Timing: before discharge  Duration: ≥ 60 minutes  N=627 | N=217 | Exclusive breastmilk feeding at discharge, and 42 days after discharge |
| **Retrospective cohort studies** | | | | | |
| Agudelo 2020 (97) | Colombia  10/2017 – 07/2018 | Inclusion criteria: Born vaginally, birth weight ≥ 2000 g, Apgar > 7, no respiratory difficulty at birth  Exclusion criteria: Resuscitation, congenital malformation, neonatal hospitalisation  GA: Preterm and term. Intervention - 39 (IQR 38.1–40.0), control – 39.0 (38.0–39.4)  BW: Intervention - 3080±360, control - 3070±400 | Timing: ≤10 minutes after birth  Duration: < 60 minutes  n=672 | Radiant heat lamp N=144 | Admission to special care nursery or neonatal intensive care nursery, special care nursery or neonatal intensive care nursery admission for hypoglycaemia |
| Bedford 2022 (98) | Canada  2008 - 2019 | Inclusion criteria: Singleton, gestation >37 weeks  Exclusion criteria: major anomalies, NICU admission > 5 days, Apgar < 3, resuscitation  GA: Term  BW: Whole cohort: Intervention – 3490±460, control -3510±500  Linked subsample: Intervention - 3540 ±480, control - 3410 ±540 | Timing: unspecified  Duration: unspecified  N= 54,475  Linked subsample n = 479 | Routine care  N= 12,072  Linked subsample n = 49 | Exclusive breastmilk feeding at discharge and at 4 months postpartum |
| Harrison 2019 (99) | USA/  Unspecified | Inclusion criteria: Term infants, congenital heart disease requiring surgical intervention within the first month of life, mother: age ≥ 18 years, English speaking.  Exclusion criteria: noncardiac congenital defects or syndromes  GA: Term  BW: Not reported | Timing: before discharge  Duration: ≥ 60 minutes  N=10 | Routine care  N=10 | Duration of initial hospital stay |
| Linares 2017 (100) | USA/  Unspecified | Inclusion criteria: Mothers: healthy, age ≥16 years, Hispanic, term infant  GA: Term  BW: Not reported | Timing: ≤10 minutes after birth  Duration: Unspecified  N=65 | Routine care N=32 | Exclusive breastmilk feeding at discharge, and at 1 month |
| Posthuma 2017 (101) | Netherlands/  08/2011 – 08/2012; 01/2013 – 12/2013 | Inclusion criteria: Caesarean section  Exclusion criteria: Preterm, general anaesthesia, fetal distress  GA: Term. Intervention - 276±8 days, control - 276±9 days  BW: Intervention - 3290±500, control - 3340±480 | Timing: > 10 minutes to 24 hours  Duration: unspecified  N=294 | N=372 | Neonatal hypoglycaemia, admission to special care nursery or neonatal intensive care nursery, hypothermia |
| Rodrigues 2006 (102) | Brazil 01/2002 – 06/2004 | Inclusion criteria: Preterm, birth weight < 2000 g, without congenital malformations, surgical intervention or record of sepsis  GA: Preterm. Intervention - 32±1.7, control - 32±2.3  BW: Intervention - 1560±220, control - 1560±250 | Timing: unspecified  Duration: ≥ 60 minutes  N=60 | Traditional method  N=60 | Duration of initial hospital stay |
| Sheedy 2022 (103) | Australia/  08/2019 – 08/2020 | Inclusion criteria: Singleton, elective caesarean section, gestation >37 weeks  GA: Term. Intervention - 38.9±0.7, control - 38.8±0.8  BW: Not reported | Timing: ≤10 minutes after birth  Duration: Unspecified  N=51 | Baby moved to postnatal ward with father/ significant other/ support person  N=51 | Exclusive breastmilk feeding at discharge (comment only) |
| Tuoni 2012 (104) | Italy/ 2006 – 2009 | Inclusion criteria: Stable, no acute illness  Exclusion criteria: Critical stages of acute pathologies, necrotising enterocolitis, chest drainage, pulmonary hypertension, vasopressor therapy, nCPAP, endotracheal intubation  GA: Preterm. Intervention - 204±16 days, control - 212±18 days  BW: Intervention - 1080±240, control - 1230±270 | Timing: Unspecified  Duration: ≥ 60 minutes  N=91 | Conventional care  N=71 | Duration of initial hospital stay |
| Vahidi 2014 (105) | Iran/ 2010 - 2011 | Inclusion criteria: Preterm, low birth weight (<1500g)  GA: Preterm. Intervention - 30.4±2.4, control - 29.6±2.2.  BW: Intervention - 1240±10, control - 1133.78±140 | Timing: Unspecified  Duration: ≥ 60 minutes  N=45 | Servo-controlled radiant warmers or cradle under hot lamps in NICU  N=45 | Duration of initial hospital stay, any breastfeeding 2 months after discharge |
| **Case-control studies** | | | | | |
| Girma 2021 (106) | Ethiopia  02/2020 – 04/2020 | Inclusion criteria: Cases: axillary body temperature >36.5°C at admission to NICU. Controls: axillary body temperature ≥36.5°C.  GA: Preterm and term  BW: Not reported | Timing: ≤10 minutes after birth  Duration: unspecified  N=73 cases  N=146 controls | No SSC | Hypothermia |
| Shibesh 2020 (107) | Ethiopia  09/2019 – 11/2019 | Inclusion criteria: Cases: axillary temperature <36.5C at admission. Controls: axillary temperature ≥36.5C at admission  GA: Preterm and term. Intervention - 36.8±3.2, control – 38.0±2.2.  BW: Intervention - 3000±610, control - 2560±1100 | Timing: unspecified  Duration: unspecified  N=129 cases  N=258 controls | No SSC | Hypothermia |
| Tasew 2018 (108) | Ethiopia  2017 | Inclusion criteria: Cases: hypothermia (< 36.5 °C) admitted to NICU, controls: no hypothermia or ≥ 36.5 °C admitted to NICU  GA: Preterm and term BW: Not reported | Timing: ≤10 minutes after birth  Duration: ≥ 60 minutes  N=88 cases  N=176 controls | No SSC | Hypothermia |

Abbreviations: GA: gestational age (weeks) mean ± standard deviation, BW: birthweight (g) mean ± standard deviation, NICU: neonatal intensive care unit; CPAP, continuous positive airway pressure, SSC: skin-to-skin contact, HIV: human immunodeficiency virus, nCPAP: nasal continuous positive airway pressure, FiO2: fraction of inspired oxygen, TORCH: toxoplasmosis, rubella, cytomegalovirus, herpes simplex or syphilis, IQR – interquartile range

1. Acharya N SR, Bhatta NK, Poudel P. Randomized control trial of kangaroo mother care in low birth weight babies at a tertiary level hospital. Journal of Nepal Paediatric Society. 2014;34(1):18-23.

2. Akcan E, Yigit R, Atici A. The effect of kangaroo care on pain in premature infants during invasive procedures. Turk J Pediatr. 2009;51(1):14-8.

3. Ali SM SJ, Sharma R, Alam S. Kangaroo Mother Care as compared to conventional care for low birth weight babies. Dicle Medical Journal/Dicle Tip Dergisi. 2009;36(3).

4. Anderson GC, Chiu SH, Dombrowski MA, Swinth JY, Albert JM, Wada N. Mother-newborn contact in a randomized trial of kangaroo (skin-to-skin) care. J Obstet Gynecol Neonatal Nurs. 2003;32(5):604-11.

5. Ayala A, Christensson K, Christensson E, Cavada G, Erlandsson K, Velandia M. Newborn infants who received skin-to-skin contact with fathers after Caesarean sections showed stable physiological patterns. Acta Paediatr. 2021;110(5):1461-7.

6. Ballesteros RM AE, Dagdag-Matias AA, Leon-Mendoza, SD. Kangaroo mother care: A randomised controlled trial on its effects on growth and stability among low birthweight infants ≤ 2000 grams in a Tertiary Government Hospital. Journal of Paediatrics and Child Health. 2012;61(1):10-9.

7. Beiranvand S, Valizadeh F, Hosseinabadi R, Pournia Y. The effects of skin-to-skin contact on temperature and breastfeeding successfulness in full-term newborns after cesarean delivery. Int J Pediatr. 2014;2014:846486.

8. Bier JA, Ferguson AE, Morales Y, Liebling JA, Archer D, Oh W, et al. Comparison of skin-to-skin contact with standard contact in low-birth-weight infants who are breast-fed. Arch Pediatr Adolesc Med. 1996;150(12):1265-9.

9. Boo NY, Jamli FM. Short duration of skin-to-skin contact: effects on growth and breastfeeding. J Paediatr Child Health. 2007;43(12):831-6.

10. Brotherton H, Gai A, Kebbeh B, Njie Y, Walker G, Muhammad AK, et al. Impact of early kangaroo mother care versus standard care on survival of mild-moderately unstable neonates <2000 grams: A randomised controlled trial. EClinicalMedicine. 2021;39:101050.

11. Bystrova K, Widstrom AM, Matthiesen AS, Ransjo-Arvidson AB, Welles-Nystrom B, Wassberg C, et al. Skin-to-skin contact may reduce negative consequences of "the stress of being born": a study on temperature in newborn infants, subjected to different ward routines in St. Petersburg. Acta Paediatr. 2003;92(3):320-6.

12. Caka SY, Topal S, Yurttutan S, Aytemiz S, Cikar Y, Sari M. Effects of kangaroo mother care on feeding intolerance in preterm infants. J Trop Pediatr. 2023;69(2).

13. Carfoot S, Williamson PR, Dickson R. The value of a pilot study in breast-feeding research. Midwifery. 2004;20(2):188-93.

14. Carfoot S, Williamson P, Dickson R. A randomised controlled trial in the north of England examining the effects of skin-to-skin care on breast feeding. Midwifery. 2005;21(1):71-9.

15. Cattaneo A, Davanzo R, Worku B, Surjono A, Echeverria M, Bedri A, et al. Kangaroo mother care for low birthweight infants: a randomized controlled trial in different settings. Acta Paediatr. 1998;87(9):976-85.

16. Charpak N, Ruiz-Pelaez JG, Figueroa de CZ, Charpak Y. Kangaroo mother versus traditional care for newborn infants </=2000 grams: a randomized, controlled trial. Pediatrics. 1997;100(4):682-8.

17. Christensson K, Siles C, Moreno L, Belaustequi A, De La Fuente P, Lagercrantz H, et al. Temperature, metabolic adaptation and crying in healthy full-term newborns cared for skin-to-skin or in a cot. Acta Paediatr. 1992;81(6-7):488-93.

18. Christensson K. Fathers can effectively achieve heat conservation in healthy newborn infants. Acta Paediatr. 1996;85(11):1354-60.

19. Chwo MJ, Anderson GC, Good M, Dowling DA, Shiau SH, Chu DM. A randomized controlled trial of early kangaroo care for preterm infants: effects on temperature, weight, behavior, and acuity. J Nurs Res. 2002;10(2):129-42.

20. Dehghani K, Movahed ZP, Dehghani H, Nasiriani K. A randomized controlled trial of kangaroo mother care versus conventional method on vital signs and arterial oxygen saturation rate in newborns who were hospitalized in neonatal intensive care unit. J Clin Neonatol. 2015;4(1):26-31.

21. Edraki M, Zendehzaban S, Beheshtipour N, Hemmati F, Haghpanah S. Comparison of the effects of attachment training for mothers on the behavioral responses of premature infants: A randomized clinical trial. Iran J Neonatol. 2015;6(2):37-42.

22. Fardig JA. A comparison of skin-to-skin contact and radiant heaters in promoting neonatal thermoregulation. J Nurse Midwifery. 1980;25(1):19-28.

23. Marin Gabriel MA, Llana Martin I, Lopez Escobar A, Fernandez Villalba E, Romero Blanco I, Touza Pol P. Randomized controlled trial of early skin-to-skin contact: effects on the mother and the newborn. Acta Paediatr. 2010;99(11):1630-4.

24. Gathwala G, Singh B, Singh J. Effect of Kangaroo Mother Care on physical growth, breastfeeding and its acceptability. Trop Doct. 2010;40(4):199-202.

25. Ghavane S, Murki S, Subramanian S, Gaddam P, Kandraju H, Thumalla S. Kangaroo Mother Care in kangaroo ward for improving the growth and breastfeeding outcomes when reaching term gestational age in very low birth weight infants. Acta Paediatr. 2012;101(12):e545-e9.

26. Gouchon S, Gregori D, Picotto A, Patrucco G, Nangeroni M, Di Giulio P. Skin-to-skin contact after cesarean delivery an experimental study. Nurs Res. 2010;59(2):78-84.

27. Hinduja AR UR. Skin to skin contact at birth for full term newborns - randomized controlled trial. Journal of Neonatology. 2014;28(10-7).

28. Kadam S, Binoy S, Kanbur W, Mondkar JA, Fernandez A. Feasibility of kangaroo mother care in Mumbai. Indian J Pediatr. 2005;72(1):35-8.

29. Kanodia P, Bora R, Gupta A. Kangaroo mother care-a cost effective and an alternate method to manage hypothermia in low birth weight babies for better clinical outcome. Value Health. 2016;19(7):A405-A.

30. KoÇ S KN. Effect of kangaroo care at birth on physiological parameters of healthy newborns. Turkish J Res Dev Nurs. 2017;19:1-3.

31. Kristoffersen L, Bergseng H, Engesland H, Bagstevold A, Aker K, Stoen R. Skin-to-skin contact in the delivery room for very preterm infants: a randomised clinical trial. Bmj Paediatrics Open. 2023;7(1).

32. Liao QH, Chen L, Fu SY, Hu HH. Effect of kangaroo mother care method on pain, growth and breastfeeding in newborns. Int J Clin Exp Med. 2020;13(6):4403-8.

33. Linner A, Kolz KL, Klemming S, Bergman N, Lillieskold S, Pike HM, et al. Immediate skin-to-skin contact may have beneficial effects on the cardiorespiratory stabilisation in very preterm infants. Acta Paediatr. 2022;111(8):1507-14.

34. Ludington-Hoe SM, Nguyen N, Swinth JY, Satyshur RD. Kangaroo care compared to incubators in maintaining body warmth in preterm infants. Biol Res Nurs. 2000;2(1):60-73.

35. Ludington-Hoe SM, Anderson GC, Swinth JY, Thompson C, Hadeed AJ. Randomized controlled trial of kangaroo care: cardiorespiratory and thermal effects on healthy preterm infants. Neonatal Netw. 2004;23(3):39-48.

36. Chi Luong K, Long Nguyen T, Huynh Thi DH, Carrara HPO, Bergman NJ. Newly born low birthweight infants stabilise better in skin-to-skin contact than when separated from their mothers: a randomised controlled trial. Acta Paediatr. 2016;105(4):381-90.

37. Mahmood I, Jamal M, Khan N. Effect of mother-infant early skin-to-skin contact on breastfeeding status: a randomized controlled trial. J Coll Physicians Surg Pak. 2011;21(10):601-5.

38. Mizuno K, Mizuno N, Shinohara T, Noda M. Mother-infant skin-to-skin contact after delivery results in early recognition of own mother's milk odour. Acta Paediatr. 2004;93(12):1640-5.

39. Mohammadzadeh A, Farhat A, Jafarzadeh M, Hasanzadeh L, Esmaeli H. Advantages of kangaroo mother care in less than 2000 grams low birth weight neonates. Medical Journal of the Islamic Republic Of Iran. 2011;25(1):11-5.

40. Mwendwa AC MR, Wamalwa DC. The impact of partial kangaroo mother care on growth rates and duration of hospital stay of low birth weight infants at the Kenyatta National Hospital, Nairobi. East African medical journal. 2012;89(2):53-8.

41. Myron MM, Garner TA, Novak CM, Koblentz AR, Kline SK. Skin-to-skin contact, the new radiant warmer. Jognn-J Obst Gyn Neo. 2017;46(3):S47-S8.

42. Nimbalkar SM, Patel VK, Patel DV, Nimbalkar AS, Sethi A, Phatak A. Effect of early skin-to-skin contact following normal delivery on incidence of hypothermia in neonates more than 1800 g: randomized control trial. J Perinatol. 2014;34(5):364-8.

43. de Ocampo FS V-UM. A randomized controlled trial of intermittent kangaroo mother care versus conventional care in increasing the rate of weight gain among low-birth-weight neonates. Acta Medica Philippina. 2021;55(9).

44. Olmedo MD, Gabas GdS, Merey LSF, Souza LSd, Muller KdTC, Santos MLdMd, et al. Physical responses of pre-term newborn babies submitted to the Kangaroo-Mother Care Method in Prone position. Fisioterapia e Pesquisa. 2012;19:115-21.

45. Pérez-Jiménez JM, Luque-Oliveros M, Gonzalez-Perez D, Rivera-Sequeiros A, Rodriguez-Blanco C. Does immediate skin-to-skin contact at caesarean sections promote uterine contraction and recovery of the maternal blood haemoglobin levels? A randomized clinical trial. Nursing Open. 2023;10(2):649-57.

46. Pouraboli B, Mahdieh E, Jahani Y. The effect of early skin to skin contact in term neonates after c-section on breastfeeding choice at the time of discharge. i-Manager's Journal on Nursing. 2019;9(2):1-8.

47. Ramanathan K, Paul VK, Deorari AK, Taneja U, George G. Kangaroo mother care in very low birth weight infants. The Indian Journal of Pediatrics. 2001;68(11):1019-23.

48. Ramani M, Choe EA, Major M, Newton R, Carlo W. Randomized trial of skin-to-skin contact to prevent hypothermia in term neonates. J Invest Med. 2015;63(2):418-.

49. Luistro CPR. Effectiveness of Kangaroo mother care in reducing morbidity and mortality among preterm neonates on RAM cannula continuous positive airway pressure: A randomized controlled trial. Current Pediatric Research. 2020;24:5-.

50. Roberts KL, Paynter C, McEwan B. A comparison of kangaroo mother care and conventional cuddling care. Neonatal Netw. 2000;19(4):31-5.

51. Rojas MA, Kaplan M, Quevedo M, Sherwonit E, Foster L, Ehrenkranz RA, et al. Somatic growth of preterm infants during skin-to-skin care versus traditional holding: a randomized, controlled trial. J Dev Behav Pediatr. 2003;24(3):163-8.

52. Sharma A. Efficacy of early skin-to-skin contact on the rate of exclusive breastfeeding in term neonates: a randomized controlled trial. Afr Health Sci. 2016;16(3):790-7.

53. Sloan NL, Camacho LW, Rojas EP, Stern C. Kangaroo mother method: randomised controlled trial of an alternative method of care for stabilised low-birthweight infants. Maternidad Isidro Ayora Study Team. Lancet. 1994;344(8925):782-5.

54. Srivastava S, Gupta A, Bhatnagar A, Dutta S. Effect of very early skin to skin contact on success at breastfeeding and preventing early hypothermia in neonates. Indian Journal of Public Health. 2014;58:22.

55. Suciu LM, Cucerea M, Simon M, Simpalean D. The effect of implementing skin-to-skin method of care for low-birth weight infants in a secondary level perinatal care center. Eur J Pediatr. 2016;175(11):1538-9.

56. Suman RPN, Udani R, Nanavati R. Kangaroo mother care for low birth weight infants: A randomized controlled trial. Indian Pediatr. 2008;45(1):17-23.

57. Thomson ME, Hartsock TG, Larson C. The importance of immediate postnatal contact: its effect on breastfeeding. Can Fam Physician. 1979;25:1374-8.

58. Thukral A, Sankar MJ, Agarwal R, Gupta N, Deorari AK, Paul VK. Early skin-to-skin contact and breast-feeding behavior in term neonates: A randomized controlled trial. Neonatology. 2012;102(2):114-9.

59. Villalon H AP. Short term effects of early skin-to-skin contact (kangaroo care) on breast feeding in healthy full term newborns. Revista Chilena de Pediatria. 1993;64:124-8.

60. Wang Y, Zhao T, Zhang Y, Li S, Cong X. Positive effects of kangaroo mother care on long-term breastfeeding rates, growth, and neurodevelopment in preterm infants. Breastfeed Med. 2021;16(4):282-91.

61. Whitelaw A, Heisterkamp G, Sleath K, Acolet D, Richards M. Skin to skin contact for very low birthweight infants and their mothers. Archives of Disease in Childhood. 1988;63(11):1377.

62. Worku B, Kassie A. Kangaroo mother care: a randomized controlled trial on effectiveness of early kangaroo mother care for the low birthweight infants in Addis Ababa, Ethiopia. J Trop Pediatr. 2005;51(2):93-7.

63. Xu J WX, Gu S, Zhang M, Cui X, Li X. Effects of kangaroo mother care during cesarean section on neonatal health parameters of late preterm newborns within" golden hour". Chinese Journal of Perinatal Medicine. 2019:560-4.

64. Zafran N, Garmi G, Abdelgani S, Inbar S, Romano S, Salim R. Impact of "natural" cesarean delivery on peripartum blood loss: a randomized controlled trial. Am J Obstet Gynecol MFM. 2022;4(4):100642.

65. Zheng Y, Xia YP, Ye WJ, Zheng CX. The effect of skin-to-skin contact on postoperative depression and physical recovery of parturients after cesarean section in obstetrics and gynecology department. Comput Math Method M. 2022;2022.

66. Bose A, Rath, K, Nayak, N. Impact of early skin-to-skin contact among cesarean section mother on breastfeeding, neonatal adaptation and maternal satisfaction. European Journal of Molecular & Clinical Medicine 2021;8(2):2021.

67. Britton GR. Early mother-infant contact and infant temperature stabilization. JOGN Nursing. 1980;9(2):84-6.

68. Kambarami RA, Chidede O, Kowo DT. Kangaroo care versus incubator care in the management of well preterm infants--a pilot study. Ann Trop Paediatr. 1998;18(2):81-6.

69. Karimi S PP, Basiri B, Roshanaei G. The effect of Kangaroo Mother Care on nutritional status and duration of hospitalization of premature infants in Iran. Journal of Postgraduate Medical Institute. 2020;34(1).

70. Kashaninia Z, Dehghan M. The effect of kangaroo care on weight gain of premature neonates in hospitalized in neonatal intensive care units. Biosciences Biotechnology Research Asia. 2015;12(2):1405-10.

71. Keshavarz M HNB. Effects of kangaroo contact on some physiological parameters in term neonates and pain score in mothers with cesarean section. Koomesh journal. 2010;11(2):91-8.

72. Kucukoglu S, Yilmaz Kurt F, Aytekin Ozdemir A, Ozcan Z. The effect of kangaroo care on breastfeeding and development in preterm neonates. J Pediatr Nurs. 2021;60:e31-e8.

73. Mathews J. A study on the effect of kangaroo mother care on neonatal temperature and weight among newborns in cuttack. Nursing Journal of India. 2018;109(6):257.

74. Mehrpisheh S, Doorandish Z, Farhadi R, Ahmadi M, Moafi M, Elyasi F. The Effectiveness of Kangaroo Mother Care (KMC) on attachment of mothers with premature infants. Eur J Obstet Gynecol Reprod Biol X. 2022;15:100149.

75. Parsa P, Karimi S, Basiri B, Roshanaei G. The effect of kangaroo mother care on physiological parameters of premature infants in Hamadan City, Iran. Pan Afr Med J. 2018;30:89.

76. Rangey PS, Sheth M. Comparative effect of massage therapy versus kangaroo mother care on body weight and length of hospital stay in low birth weight preterm infants. Int J Pediatr. 2014;2014:434060.

77. Obaid Ur Rehman M, Hayat S, Gul R, Irfan Waheed KA, Victor G, Khan MQ. Impact of intermittent kangaroo mother care on weight gain of neonate in nicu: Randomized control trial. J Pak Med Assoc. 2020;70(6):973-7.

78. Safari K, Saeed AA, Hasan SS, Moghaddam-Banaem L. The effect of mother and newborn early skin-to-skin contact on initiation of breastfeeding, newborn temperature and duration of third stage of labor. Int Breastfeed J. 2018;13:32.

79. Solanki S, Kachhiya P, Patel K, Patel V, Baria T, Jatva K, et al. A study to evaluate effectiveness of kangaroo mother care on physiological parameters of premature babies in Vadodara City. Indian Journal of Forensic Medicine & Toxicology. 2021;15(4):2444-7.

80. Swarnkar K, Vagha J. Effect of kangaroo mother care on growth and morbidity pattern in low birth weight infants. J Krishna Inst Med S. 2016;5(1):91-9.

81. Toprak FU, Erenel AS. The effect of kangaroo care practice after caesarean section on paternal-newborn interaction: A mixed-methods study in Turkey. Midwifery. 2022;115:103489.

82. Albuquerque RSd, Mariani Neto C, Bersusa AAS, Dias VM, Silva MIMd. Newborns' temperature submitted to radiant heat and to the Top Maternal device at birth. Revista Latino-Americana de Enfermagem. 2016;24.

83. Ferrara P, Spina G, Cutrona C, Romano V, Andrissi L, Farchi F, et al. Skin-to-skin contact: an easily implemented intervention to reduce perinatal complications and pain perception in a rural African community. Minerva Pediatr. 2017;69(5):453-4.

84. Gonuguntla Y, Metgud T, Mahantshetti NS. Effectiveness of kangaroo mother care in the management of twin low-birth-weight neonates: A non-randomized, open, controlled trial. Iran J Neonatol. 2018;9(3):6-13.

85. Kristoffersen L, Stoen R, Hansen LF, Wilhelmsen J, Bergseng H. Skin-to-skin care after birth for moderately preterm infants. Jognn-J Obst Gyn Neo. 2016;45(3):339-45.

86. Li W, Yu Z, Jing Y. Effect evaluation of kangaroo mother care in Liping area, Guizhou province,China. BMC Pediatrics. 2022;22(1):649.

87. Shattnawi KK, Al-Ali N. The effect of short duration skin to skin contact on premature infants' physiological and behavioral outcomes: A quasi-experimental study. J Pediatr Nurs. 2019;46:e24-e8.

88. Y Y. Influence of kangaroo care and traditional nursing care on premature physiologic parameters. Nurs Res. 2000;8:362-74.

89. Casolino M DMS. Skin to skin contact after birth: Prevalence and duration of maternal breastfeeding and prevalence of functional gastrointestinal disorders. Journal of Pediatric Gastroenterology and Nutrition. 2022;74:402.

90. Guala A, Boscardini L, Visentin R, Angellotti P, Grugni L, Barbaglia M, et al. Skin-to-skin contact in cesarean birth and duration of breastfeeding: A cohort study. ScientificWorldJournal. 2017;2017:1940756.

91. Juan J, Zhang XS, Wang XY, Liu J, Cao YL, Tan L, et al. Association between skin-to-skin contact duration after caesarean section and breastfeeding outcomes. Children-Basel. 2022;9(11).

92. Lamy F, da Silva AAM, Lamy ZC, Gomes MASM, Moreira MEL, Canguru GAM, et al. Evaluation of the neonatal outcomes of the kangaroo mother method in Brazil. J Pediat-Brazil. 2008;84(5):428-35.

93. Mikiel-Kostyra K, Mazur J, Boltruszko I. Effect of early skin-to-skin contact after delivery on duration of breastfeeding: a prospective cohort study. Acta Paediatr. 2002;91(12):1301-6.

94. Ruiz NM, Iniguez JPG, Gracia SR, Villagrasa MPS. Prospective study on influence of perinatal factors on the development of early neonatal hypoglycemia in late-preterm and term infants. An Pediatr. 2022;96(3):230-41.

95. Suzuki S. Effect of early skin-to-skin contact on breast-feeding. J Obstet Gynaecol. 2013;33(7):695-6.

96. Zhang B, Duan Z, Zhao Y, Williams S, Wall S, Huang L, et al. Intermittent kangaroo mother care and the practice of breastfeeding late preterm infants: results from four hospitals in different provinces of China. Int Breastfeed J. 2020;15(1):64.

97. Agudelo S, Diaz D, Maldonado MJ, Acuna E, Mainero D, Perez O, et al. Effect of skin-to-skin contact at birth on early neonatal hospitalization. Early Human Development. 2020;144.

98. Bedford R, Piccinini-Vallis H, Woolcott C. The relationship between skin-to-skin contact and rates of exclusive breastfeeding at four months among a group of mothers in Nova Scotia: a retrospective cohort study. Can J Public Health. 2022;113(4):589-97.

99. Harrison TM, Chen CY, Stein P, Brown R, Heathcock JC. Neonatal skin-to-skin contact: Implications for learning and autonomic nervous system function in infants with congenital heart disease. Biological Research for Nursing. 2019;21(3):296-306.

100. Linares AM, Wambach K, Rayens MK, Wiggins A, Coleman E, Dignan MB. Modeling the influence of early skin-to-skin contact on exclusive breastfeeding in a sample of hispanic immigrant women. J Immigr Minor Health. 2017;19(5):1027-34.

101. Posthuma S, Korteweg FJ, van der Ploeg JM, de Boer HD, Buiter HD, van der Ham DP. Risks and benefits of the skin-to-skin cesarean section - a retrospective cohort study. J Matern Fetal Neonatal Med. 2017;30(2):159-63.

102. Rodrigues MAG CM. Trial gain of weight and hospital length stay of the low birth weight preterm infant in assistance for kangaroo mother care. Revista Eletronica de Enfermagem. 2006;8(2):185-91.

103. Sheedy GM, Stulz VM, Stevens J. Exploring outcomes for women and neonates having skin-to-skin contact during caesarean birth: A quasi-experimental design and qualitative study. Women and Birth. 2022;35(6):e530-e8.

104. Tuoni C, Scaramuzzo RT, Ghirri P, Boldrini A, Bartalena L. Kangaroo mother care: four years of experience in very low birth weight and preterm infants. Minerva Pediatrica. 2012;64(4):377-83.

105. Vahidi RG, Gholipour K, Jannati A, Hosseini MB, Ghoddoosi-Nejad J, Bayan H. Cost and effectiveness analysis of kangaroo mother care and conventional care method in low birth weight neonates in Tabriz 2010-2011. J Clin Neonatol. 2014;3(3):148-52.

106. Girma B, Tolessa BE, Bekuma TT, Feyisa BR. Hypothermia on admission to a neonatal intensive care unit in Oromia, western Ethiopia: a case-control study. BMJ Paediatr Open. 2021;5(1):e001168.

107. Shibesh BF, Yalew WA, Beyene MB, Minyiwab GW. Determinants of neonatal hypothermia among neonates admitted to neonatal intensive care unit northwest, Ethiopia, case-control study. J Matern-Fetal Neo M. 2022;35(20):3903-8.

108. Tasew H, Gebrekristos K, Kidanu K, Mariye T, Teklay G. Determinants of hypothermia on neonates admitted to the intensive care unit of public hospitals of Central Zone, Tigray, Ethiopia 2017: unmatched case-control study. BMC Res Notes. 2018;11(1):576.
