# Supplementary material for: Skin-to-skin contact for the prevention of neonatal hypoglycaemia: a systematic review and meta-analysis
Source: BMC Pregnancy Childbirth. 2023 Oct 21;23:744. doi: 10.1186/s12884-023-06057-8 (PMC10590034; doi:10.1186/s12884-023-06057-8)
Supplement: Supplementary file 5 — Additional file 5. Funnel plots [file 12884_2023_6057_MOESM5_ESM.docx]

Funnel plots and Egger’s test result

1. Duration of initial hospital stay


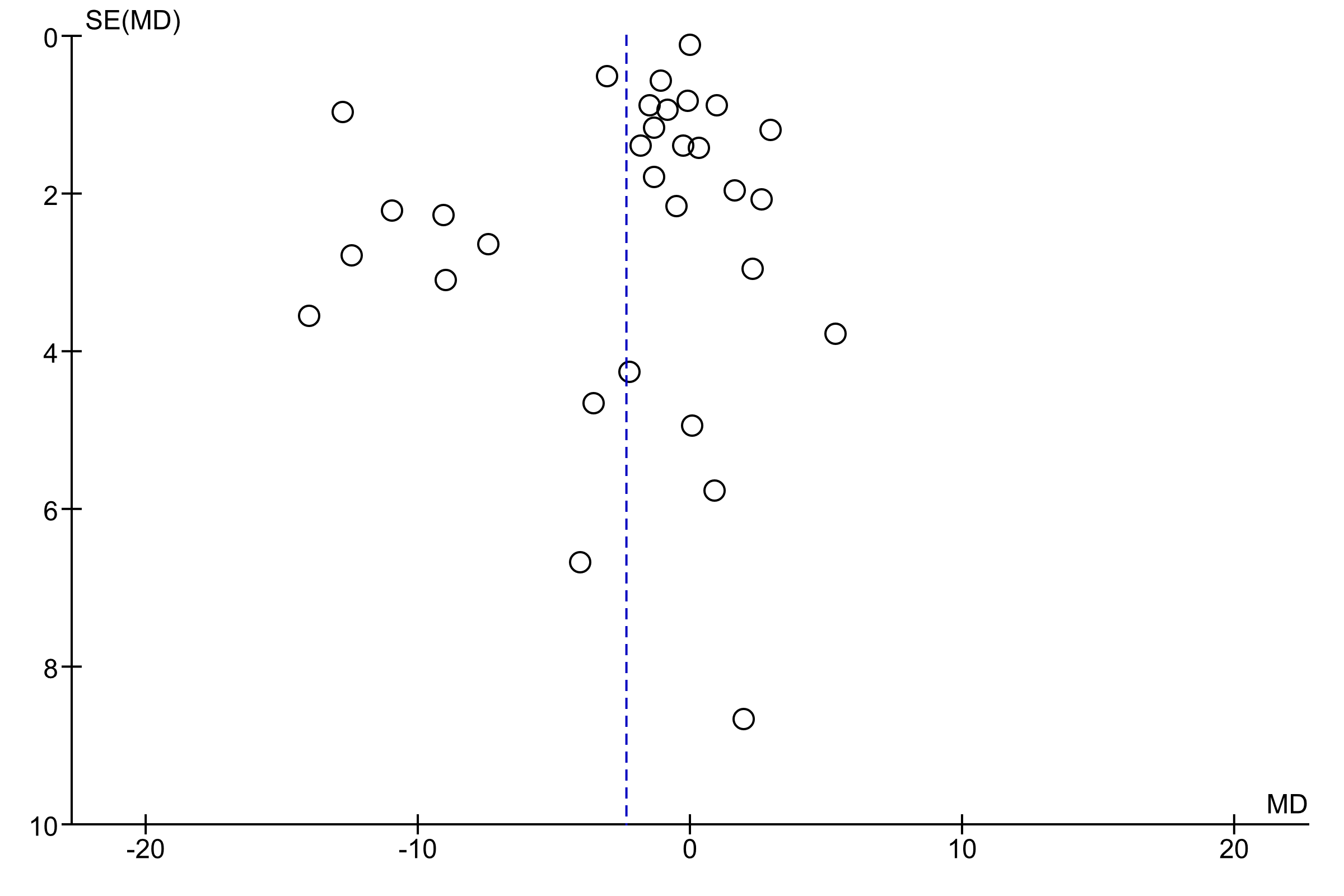


p-value = 0.0159

2. Hypothermia


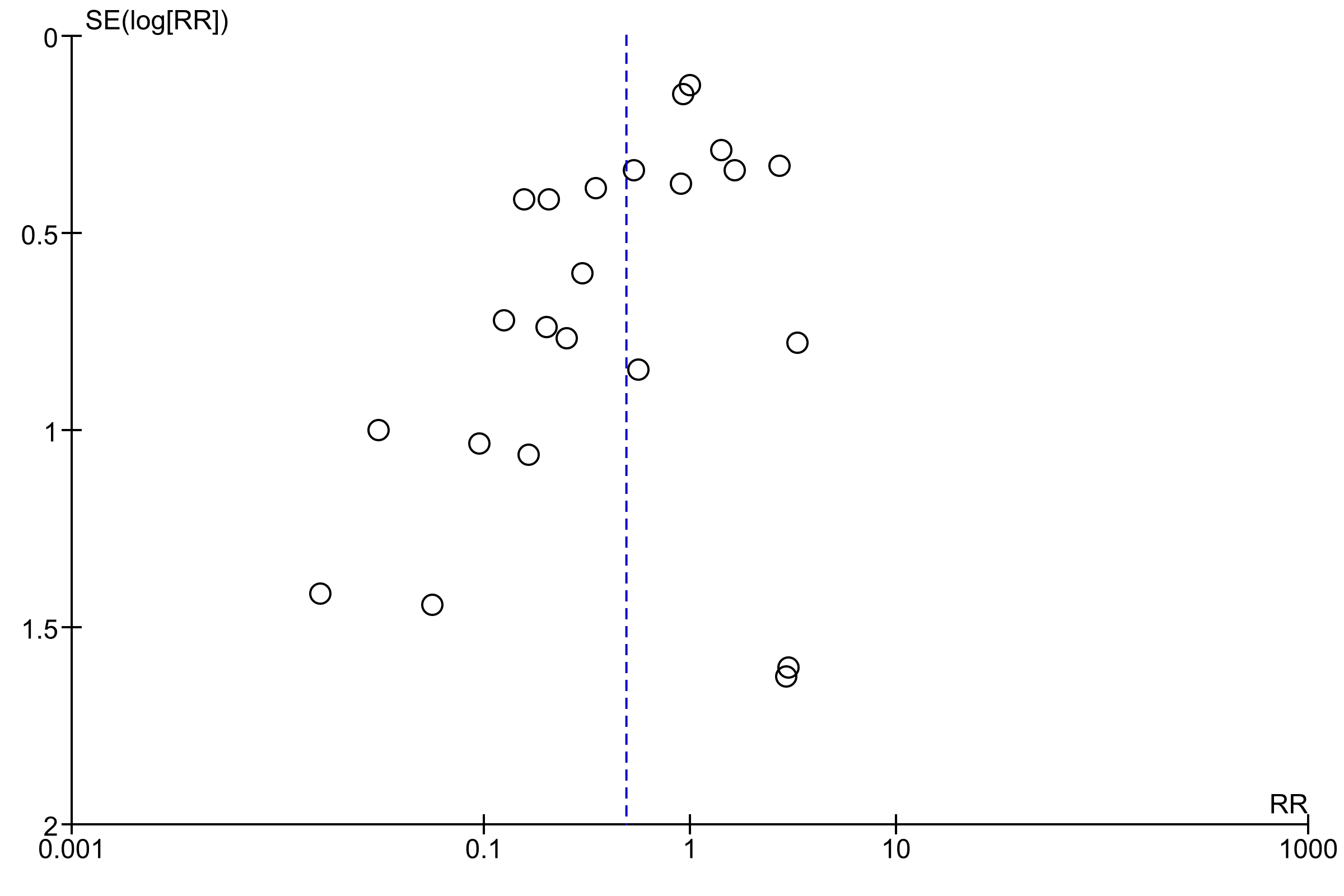


p-value = 0.0257

3. Exclusive breastmilk feeding at discharge


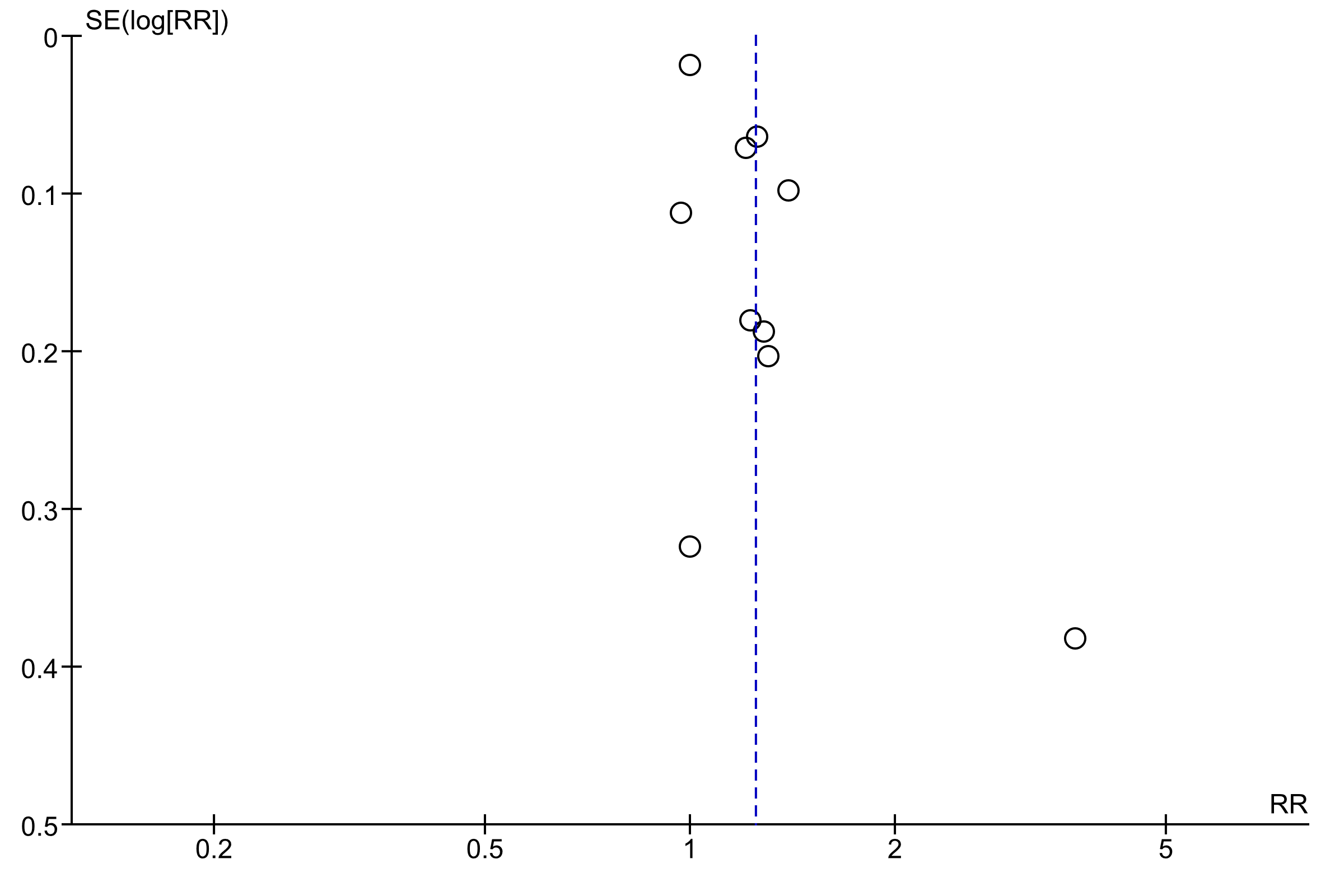


p-value = 0.0160

4. Exclusive breastmilk feeding from discharge – 3 months


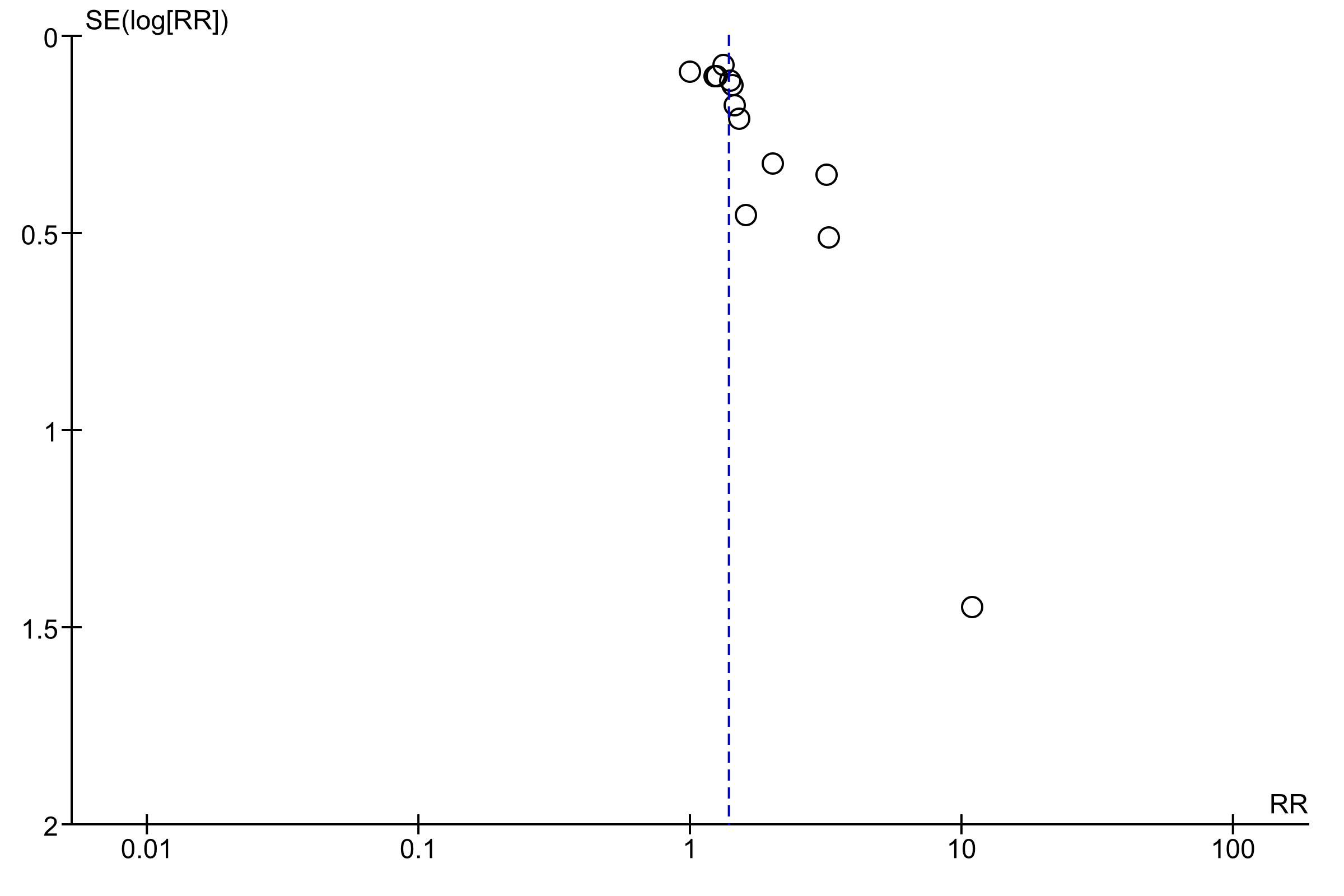


p-value = 0.0018
